# Changes in value priorities due to the COVID-19 pandemic—A 4-year cross-sectional study with German students

**DOI:** 10.1371/journal.pone.0297236

**Published:** 2024-01-19

**Authors:** Christian Hannes, Sarah Schiffer, Rüdiger von Nitzsch

**Affiliations:** Decision Theory and Financial Services Group, RWTH Aachen University, Aachen, Germany; University of Potsdam: Universitat Potsdam, GERMANY

## Abstract

In March 2020, the WHO declared the coronavirus a pandemic. Since then, the German government has tried to control the spread of the virus with various restrictions. These restrictions had a direct impact on the life of German students. In this study, we investigate to what extent the restrictions led to a change of value priorities of German students. From January 2019 to January 2022, we conducted a cross-sectional study with four measurement points and, in total, 1,328 participants. Two measurement points were before the first outbreak of COVID-19 in Germany, one in the second lockdown phase and the third after two years in the pandemic. In this study, the students were asked to indicate their value priorities while solving a real-world decision problem important to them. Results suggest increased value priorities of the values *Intellectual Fulfillment* and *Environment and Nature* and a decrease of *Family and Partner* value priority as a direct effect of the second lockdown phase. We also found small differences regarding value priorities between the male and female subjects. The data show bounce-back effects as the pandemic became more normal to the students. In the long run, value priorities seem to be stable, with the exception of a longer-lasting increase in *Freedom and Independence*.

## Introduction

The current COVID-19 pandemic poses a major threat to physical and mental health. It has severely affected people’s lives worldwide for more than two years. Especially at the beginning, the images in the news (e.g., from Bergamo in Italy) caused concern and fear. Governments worldwide were challenged to respond quickly and efficiently to this threat. Therefore, several governments initiated lockdowns to protect the population, bringing public life to a standstill and limiting personal contact. The resulting social distancing and loneliness also negatively impacted many people’s mental health affecting all age groups and population segments [1]. This pandemic, as an incisive event, provides an opportunity for various scientific investigations, including behavioral research [2, 3]. Several studies looked at extreme behavior, such as panic hoarding, especially the struggle for toilet paper [4, 5]. For some people, the pandemic caused long-term behavioral changes, e.g., anxiety disorders, depression, and sleep disorders [6].

As an extreme event, the pandemic also offers interesting research opportunities for examining the stability of people’s personal values in times of crisis. Personal values are generally considered relatively stable over a lifetime [7]. However, repeated priming, actively reconsidering one’s values, or extreme situations can cause changes in the value system [8, 9]. Investigating these effects is important in our field of decision research since decisions and values are closely related: values form the basis for identifying the goals a person strives for and therefore function as guidelines for motivated action [10, 11].

Initial studies suggest that the COVID-19 pandemic, as an extreme event, shifts individuals’ value systems [12–16]. All studies claim they cannot generalize their implications as they focus on people from a specific country, and the pandemic hit countries differently. More data is needed to better understand the changes in individuals’ value systems caused by the pandemic, better assess discrepancies between study results, and draw firm conclusions [16]. Existing studies recommend looking at other countries, a longer period before and after the outbreak of the pandemic [13], and using other data collection methods such as reports on social values [14] or the lexical analysis of texts in newspapers [17]. The latter is addressed in a study by van de Poel et al. [15]. Results of a study conducted in Poland also indicate that gender differences regarding the negative effect of the lockdown on overall well-being exist. Still, again, these cannot be generalized and therefore need further research [13].

Our study contributes to the research gap by addressing country-specific differences, taking a longer-term view before and after the coronavirus outbreak, and looking at possible gender differences. Therefore, in our cross-sectional study from 2019 to 2022, we investigate whether and to what extent changes can be observed within the personal value system of German students in the context of the COVID-19 pandemic and whether there are differences to the results in Poland and Australia. Our contribution not only helps to better understand the psychological consequences of the lockdowns and their differences in various countries. We furthermore test if the reported gender differences can also be found in our data. Moreover, we use a more hands-on research method to derive data. We evaluate changes in the value priorities of Germans regarding twelve values that are important for personal decisions and collect data about value priorities from real-life decisions with a decision support system.

The structure of this paper is the following: The first sub-sections outline the construct of value systems, a model of value change, and the results of value research in the context of the coronavirus pandemic. Based on this knowledge, we present our hypotheses in the corresponding sub-section. Using data collected from 2019 to 2022, we examine the differences in values at four measurement points and check our hypotheses in the results section. By looking at multi-year data, we expect indications of the stability of values and whether the pandemic has spurred longer-term value changes.

### Human values

There are many different definitions of values. Kluckhohn defined values as „a conception, explicit or implicit, distinctive of an individual, or characteristic of a group, of the desirable which influences the selection from available modes, means and ends of action”[18]. He distinguished between two types of personal values: implicit values, which are not observable, and explicit values, which a person attributes to themself. This differentiation is also found in the concept of motives [19]. In the literature, values and motives are sometimes used synonymously (and equally synonymously with needs, desires, attitudes, preferences, objectives, norms, and virtues) since the boundaries between the individual constructs are blurred [20–22]. Nevertheless, these are different concepts [23].

Besides the existence of different definitions of the term value one can also distinguish between several types of values. Kluckhohn himself described two types of values: values of an individual and values of a group. Rohan [22] proposed to differentiate between two types of “intrapsychic value systems” that are located within a person (internal values)–the “personal value system” that includes the own (implicit or explicit) values of a person and several “social value systems” that refer to others’ expectations–and on top of that certain “ideological value systems” regarding value priorities promoted by groups (for example religious congregations, societies, cultures). The latter is not located within a person (external values). Each value system includes a finite number of universally important values, with people differing in the relative importance they place on these values.

The internal values are shaped by the external values in that they are learned through upbringing, training, contact, etc. [24]. It can be assumed that internal values ​​are formed in children and young people over time. From adulthood onward, internal values can then be described as consolidated and largely stable [25]. Allport stated: „Personal values are the dominating force in life, and all of a person’s activity is directed toward the realization of his values”[10]. Personal values can thus be understood as an overarching concept shaping people’s motives and guiding their decisions and actions (motivated action as an interaction of person and situation) [19]. Consequently, they are an integral part of decision-making processes and the justification of decisions [26, 27]. In this paper, we refer to these “personal values” (of the personal value system), precisely the self-reported explicit personal values of a single person that determine what they consider important in their life [28] and which Schwartz defines as: “broad desirable goals that motivate people’s actions and serve as guiding principles in their lives” [11].

### Value systems

Rokeach dealt extensively with personal values and developed the Rokeach Values Survey (RVS) with 18 fundamental and 18 instrumental values [25]. Another instrument is the List of Values, based on Rokeach’s explanations and insights of Feather [29] and Maslow [30]. In this list, Rokeach’s fundamental values were modified and reduced to 9 core values: self-respect, security, warm relationships with others, sense of accomplishment, self-fulfillment, sense of belonging, being well respected, fun and enjoyment, and excitement [31]. As mentioned above, values can be arranged in a so-called value system [22], and the best-known value system was developed by Schwartz [32]. Schwartz [33] ranks values according to the degree of compatibility of the objectives pursued by the values. He uses two bipolar dimensions to illustrate the "motivational goals" behind the values.

The first dimension differentiates between the opposing poles of Openness to Change and Conservatism. This dimension addresses the conflict between values that focus on independent thinking, acting, and feeling, readiness for change (self-direction, stimulation), and values that reflect the structure, discipline, preservation of the past, and resistance to change (security, conformity, tradition). The second dimension differentiates between Self-Enhancement and Self-Transcendence, contrasting the conflict between values focusing on the pursuit of self-interest, relative success, and relative power over others (power, achievement) and values focusing on the welfare and interests of others (universalism, benevolence). The individual values in Schwartz’s model are not to be understood as concrete entities but as levels on a continuum [34]. Therefore, Schwartz arranges them in a circular shape, with values with a high potential for conflict having a large distance in the circle. This, in turn, means that people who prioritize the values of one dimension particularly strongly usually weight the opposite values lower. Originally Schwartz defined ten values [35, 36], which are still considered a reference in many studies today. In the meantime, a second, extended version with 19 values also exists [37]. The assumption behind the value system is that the values of all people can be covered by it. Individuals, therefore, do not differ in the number of their values but in prioritizing the respective values (S1 Fig).

Our study examines changes in value priorities for twelve personal values that are particularly important for private decisions. Table 1 shows our 12 values and how they relate to the corresponding values according to Schwartz. In this way, our results can be better compared to existing knowledge.

**Table 1:** Values used in this study and their corresponding value in Schwartz’s value system.

Values used in this study	Corresponding value at Schwartz	Schwartz’s Value Definition
Family and Partner	Benevolence	“Preserving and enhancing the welfare of those with whom one is in frequent personal contact (the ‘in-group’). […] Most critical are relations within the family and other primary groups.” [36]
Friends and Social Relations	Benevolence	
Justice and Fairness	Universalism	“Understanding, appreciation, tolerance, and protection for the welfare of all people and for nature. […] People may […] realize that failure to accept others who are different and treat them justly will lead to life-threatening strife.” [36]
Environment and Nature	Universalism	
Intellectual Fulfilment	Self-direction	“Independent thought and action—choosing, creating, exploring. Self-direction derives from organismic needs for control and mastery […] and interactional requirements of autonomy and independence” [36]
Freedom and Independence	Self-direction	
Excitement and New Experiences	Stimulation	“Stimulation values derive from the organismic need for variety and stimulation” [36]
Competence	Achievement	“Defining goal: personal success through demonstrating competence according to social standards.” [36]
Power and Leadership	Power	“Defining goal: social status and prestige, control or dominance over people and resources.” [36]
Wealth	Power	
Financial Security	Security	“Defining goal: safety, harmony, and stability of society, of relationships, and of self.” [36]
Honesty and Ethics	Conformity	“Conformity values derive from the requirement that individuals inhibit inclinations that might disrupt and undermine smooth interaction and group functioning” [36]

### Model of value change

Value priorities are considered relatively stable [25] and are rarely subject to sudden and sustained change [38]. Nevertheless, researchers have long been concerned with the issue of changing values because values impact people’s decision-making behavior [39]. For example, personal values have been found to develop at a young age and change throughout life. It is found that older people tend to prioritize values from the Conservation and Self-Transcendence dimensions, while younger people often prioritize Self-Enhancement and Openness to Change [39, 40]. The stability of value priorities also changes with age. According to the Aging-Stability Hypothesis, value priorities become more stable the older the individual is [41]. However, some studies (e.g., [42–44]) contradict this and show remarkable changes in value priorities at older ages.

The question of what triggers a change in values is particularly interesting. Bardi and Goodwin [9] provide a theoretical model that explains the process of individual value change (S2 Fig).

This model describes two ways to change value priorities: the "automatic" and the "effortful" way. At the beginning of both paths, an external event ("environmental cues") initiates a change in value priorities. A value must be primed to activate the "automatic" path. Various environmental factors can prime the person’s values, for example, hearing a language associated with specific values or visualization in the form of posters, photos, etc., in which a situation that addresses a particular value is depicted. The subconsciously activated value can now lead to an initial change in value priorities. At the beginning of the "effortful" path is the conscious re-evaluation of the value priorities. The re-evaluation can be started by explicitly asking people about their value priorities, for example. The reconsideration of a person’s value priorities can lead to an initial change in values, just as in the “automatic” path. However, such one-time influences only lead to short-term changes in value priorities [11].

According to the model, values must be primed repeatedly (e.g., listening to the language associated with specific values every day) or value priorities need to be re-evaluated repeatedly to achieve a long-term change in value priorities. Only through regular activation is the particular value reinforced in the value system, and the person’s thinking and actions become more aligned. As a result, the person changes their thought pattern to fit the new value priorities, and the value change persists in the long run.

In Addition, Bardi and Godwin [9] point out that even a single, highly influential event can permanently change value priorities. Sagiv and Schwartz [11] also emphasize that major life events can lead to substantial changes in value priorities. In this case, the event must be so substantial or inciting for a person that the person deeply examines their values. Examples of a change in value priorities as a result of an extreme event are, for instance, the birth of a child and the resulting responsibility as a parent [45], the financial crisis in 2008 [46], and terrorist attacks, such as the attack on the World Trade Center in 2001 [47], or wars [48]. Due to such drastic events, either values can be reprioritized in the long term, or they can change only temporarily. The temporary change can last for different lengths of time. Verkasalo et al. report a return to original value levels between eleven days and five months and call this the “bounce-back effect” [47].

Bardi and Goodwin’s model shows one way to resolve the discrepancy between the aging-stability hypothesis and a substantial change in values in old age. On the one hand, the stability of value priorities in old age can be explained by the fact that priorities are "strongly crystallized" over time [49]. On the other hand, drastic life changes play a significant role in the studies mentioned above [42–44].

### Value change due to the corona virus pandemic

The coronavirus pandemic represents an extreme event that has a global impact on human life. Therefore, it is reasonable to assume that this event is also associated with a change in value priorities. Initial studies have already investigated the impact of the coronavirus pandemic on value systems: Bojanowska et al. [13] considered how value priorities and well-being changed as a result of the imposition of the first lockdown in Poland. To do so, they asked adults in Poland about their value priorities (Schwartz’s 19 values Portrait of Values Questionnaire) nine months before the first lockdown in Poland (first time point) and two (second time point) and four weeks (third time point) into the first lockdown which was announced on March 13, 2020. Their study is part of a longitudinal project on values and well-being, which they had started before the pandemic. They found that self-direction (thought), conformity (rules), humility, and universalism (nature and tolerance) were prioritized more strongly at time points two and three than before, while hedonism declined in importance. In contrast, the values (personal and social) security, interpersonal conformity, caring, and universalistic concern were temporarily more prioritized at time point two but converged to the initial level at time point three. Daniel et al. [14] analyzed adults’ values in Australia (“Best-Worst Refined Value scale”). They compared data from five surveys (three pre-pandemic in 2017–2019; the fourth in April 2020; and the fifth in late 2020). They found that conservation values (order and stability) were more prioritized in the pandemic. In contrast, openness to change values (*self-direction* and *stimulation*) became less important at the pandemic’s beginning, with this effect leveling off by the end of 2020. Self-transcendence values (care for close others, society and nature) were less prioritized at the end of 2020. The effects were more pronounced for individuals with stronger fears regarding the pandemic.

Vecchione [16] investigated the shift in values during the coronavirus pandemic in Italy, a severely affected country, especially in the beginning. He examined how Schwartz’s higher-order values changed between the summer of 2020 and November 2020. The results showed no mean-level change in the four higher-order groups of Schwartz’s two dimensions but rather significant inter-individual differences in the extent of change with economic status as a significant predictor of change in conservation values. Contrary to these results, Potocan and Nedelco [12], who analyzed value change in Slovenia, report a decrease in mean-level for all four higher-order groups of Schwartz personal values during the lockdown, followed by a bounce-back effect. Van de Poel et al. [15] employ a different method in their study. Using topic modeling, they analyzed COVID-related news articles from 2016 to 2020 from six countries for changes in the frequency with which those news articles addressed eleven different values. They report that the first moths of the pandemic led to a punctuated change in social values. In particular, it was observed that in the first few months, the values ​​of safety and health appeared more frequently, while democracy, privacy, and socio-economic equality decreased significantly. This observation was also followed by a bounce-back effect [15].

### Hypotheses

The preceding discussion has shown that extreme events can trigger a change in value prioritization among individuals, especially for those values strongly addressed by the event. Based on these insights gained, we formulate hypotheses about the value changes that might have arisen among German students due to the Corona pandemic in the following sections.

The severe restrictions of social interaction may be a starting point for changes in value priorities. For example, Bojanowska et al. [13] report that the pandemic situation in Poland increased the value priorities of Benevolence, Universalism, and Self-direction—Thought. Benevolence describes the value of prioritizing the welfare of people with frequent and close contact. Due to the contact restrictions in Germany, interaction has been limited to a small group of people. Family and partners were seen frequently, but relationships with friends and other contacts were more challenging to maintain [50]. People may have become aware of the lack of social relations with friends and reconsidered their values (effortful way of value change) as they started to miss their friends and other social relations. Therefore, we suspect that the *Friends and social relations* value will increase due to the lack of social relationships with (distant) friends and casual acquaintances.

**H1: The value priority of *Friends and social relations* will increase during the pandemic.**

Students have often been at their parent’s homes due to the lack of presence at the university. Furthermore, they have digitally participated in their studies. Therefore, many students’ contact with family and partners intensified. The need for family and partner thus has been fulfilled substantially or even over-fulfilled, especially during curfews and contact restrictions. Students got primed (automatic way) about the value of *Family and Partner* every time they saw each other. In this case, priming is not leading to an increase in the value as the need for family is overwrought, but it can lead to a loss of strength of the individual need for a certain time [51, 52]. Therefore, we assume that this value priority decreases.

**H2: The value priority of *Family and Partner* will decrease during the pandemic.**

The lockdowns triggered by the pandemic caused people in Germany to spend much more time in nature [53, 54]. In some cases, nature was the only place besides one’s home where people were allowed to spend time without personal restrictions. Being in nature can prime (automatic way) people to prioritize this value. Moreover, some people may be triggered by their time in nature and start thinking about how important nature and environment are for them (effortful way). As people felt safe and free in nature, we expect (in accordance with the results of Bojanowska et al. [13] for Poland) an increase in this value priority.

**H3: The value priority of *Environment and Nature* will increase during the pandemic.**

The contact and movement restrictions reduced the ability to move freely in society. People in Germany became aware of this lack (automatic way) and therefore increased their priority for *Freedom and Independence*. At the same time, there was a major debate about the appropriateness of measures to restrict the virus [55]. This debate may have led to a conscious reconsideration (effortful way) of their *Freedom and Independence* value priority. Due to unreliable data, people in Germany could form their own opinions on the subject while at the same time allowing for other views. Developing one’s opinion became an important issue [56]. As the restrictions severely affected people, they wanted to understand why these restrictions were needed. The wish to understand the current situation may have primed the value of Intellectual Fulfillment (automatic way). Bojanowska et al. [13] also identified a rise in these value priorities in Poland. We, therefore, assume that the value priority for the values of *Freedom and Independence*, and *Intellectual Fulfillment* increases.

**H4a: The value priority of *Freedom and Independence* will increase during the pandemic.**
**H4b: The value priority of *Intellectual Fulfillment* will increase during the pandemic.**

For the value priorities *Competence*, *Excitement and New Experiences*, *Power and Leadership*, *Honesty and Ethics*, and *Justice and Fairness*, *Financial Security* and *Wealth*, we do not expect any significant changes in connection with COVID-19 because these values were not significantly addressed by the pandemic among German students: Thus, while students’ learning situation has changed, they have still been challenged to demonstrate their *Competence* in exams now administered digitally. And while student life has been severely curtailed, and it can be argued that this has made *Excitement and New Experiences* less possible, the lockdowns have also offered new challenges and new types of experiences. The role of a strong leader has become more important during the pandemic [57], and in principle, it is possible that this also influences the personal value of *Power and leadership*. However, the majority of students are not entrusted with a leadership role, so the participants of our study do not directly address this value. *Honesty and Ethics*, as well as *Justice and Fairness*, are values ​​that relate to behavioral expectations toward others. However, the pandemic as an extreme event was not triggered by people (unlike war, for example) but by a virus to which the whole world was helplessly exposed. Therefore, these values were not directly affected. In general, studies on value change due to extreme events indicate that the value priority of *security* often increases as subjects find themselves in a more uncertain situation. Uncertainty has been described as financial uncertainty during the financial crisis [46] and health uncertainty during the World Trade Center attack [47]. In the context of the coronavirus pandemic and the first lockdown in Poland, Bojanowska et al. [13] also reported an increase in the value priority of security, but in this case, only in the short term. In their evaluation, they found that the value priority *Security* significantly increased two weeks after the lockdown, while this effect weakened again after four weeks. Students in Germany receive alimony or student loans and thus a secure income. Financial losses could occur during the pandemic due to the loss of part-time jobs, e.g., in the catering industry. At the same time, however, the lockdown reduced the usual expenses as parties were banned and restaurants and cultural venues were closed. This means that for many students, the lockdown has not resulted in any significant changes in assets that could have led to a revision of their personal values. A study conducted in the USA [58] confirms our assumption that wealth distribution was largely stable during the pandemic. For these reasons, we do not expect any significant changes in the value priorities *Financial Security* and *Wealth*.

Finally, in accordance with the results of Bojanowska et al. [13] for Poland, Potocan and Nedelko [12] for Slovenia, and Daniel et al. [14] for Australia, we expect that the Corona-Pandemic will lead to mainly short-time changes in value priorities. Because according to the model of value change in 2.2, change in value priorities needs repeated interactions to manifest in a long-term effect. Regarding COVID-19, the triggers of the changes in priorities are mainly the consequences of restrictions to reduce the spread of the virus. But these restrictions were only temporary in Germany, especially during two lockdowns. However, a weakening or the complete elimination of the restrictions means that “the trigger will fall away”.

## Methods

### Procedure and participants

In our study, subjects run through a decision-making process relevant to them in their professional context with the help of the Entscheidungsnavi [59]. The Entscheidungsnavi (www.entscheidungsnavi.de) is a decision support system that supports users in reflective decision-making in a five steps process. It combines Keeney’s value-focused thinking approach [60] with various problem structuring and debiasing methods. Keeney argues that to improve decision quality, people should be more concerned with personal values when making decisions because each alternative in a decision context is a means of realizing one’s values [61]. Therefore, in his value-focused thinking approach, he recommends that the process of decision-making should be directly aligned with personal values. People should start the process by thinking about what is important in their situation before they consider how to achieve the desired.

The success of the value-focused thinking approach has been proven in many studies. In their work, Siebert and Keeney [62] show that more and better options for action can be generated with the help of this approach than with conventional approaches. A comprehensive overview of further studies on value-focused thinking can be found in Parnell et al. [63]. For this reason, in the Entscheidungsnavi, users must also reflect on their values and identify their "objectives”.

The tool actually goes one step further than Keeney, who defines values and objectives differently but does not ask for them separately in the process step [60]. In the first step of the decision-making process, the participants define their decision situation. In the spirit of value-focused thinking [60], subjects are encouraged to reflect on their personal values and motives. For this purpose, the Entscheidungsnavi provides a list of the twelve personal values we mentioned above and several motives for self-assessment. The development team of the Entscheidungsnavi elaborated on this list in a brainstorming session with several people. Since motives, unlike values, are situational, they are irrelevant to this study and, therefore, not considered in detail. After reflecting on personal values and a final decision statement, the participants work out their objectives within the decision situation, systematically develop alternatives for action, and evaluate these with the help of the objectives in a consequences table. At the end of the decision-making process, the Entscheidungsnavi ranks the alternatives based on the respective utility values. The Entscheidungsnavi calculates utility values based on the user’s risk assessments in the consequences table. The participants can then reflect on their results and adjust any deficiencies in the decision model (S3 Fig).

The study participants were students of the course "Decision Theory" at RWTH Aachen University. As part of the lecture, the students had the opportunity to systematically work out a complex decision that was important to them by using the Entscheidungsnavi and receive a bonus on their course grade if they worked on it appropriately. In principle, personal decisions, socio-political issues, or business topics could be considered for this purpose. Our study only assesses data sets on personal career decisions where intrinsic personal values are directly relevant. Students could hand in their work within the decision support system. Therefore, they hey had to create an account within the system. While doing so, they could check a box to allow us to use their data for scientific purposes. The exact statement is: „I hereby agree that the data entered by me may be used anonymously for scientific purposes and to improve the tool”. Awarding bonuses for handing in voluntary work that helps students deepen their knowledge from the lecture is customary at our university and systematically approved by the study and examination regulations. Using data from voluntary work for research is only permitted at RWTH Aachen University when there is an opt-in procedure, and students can also get a bonus when they disagree on sharing their data.

**Fig 1:**
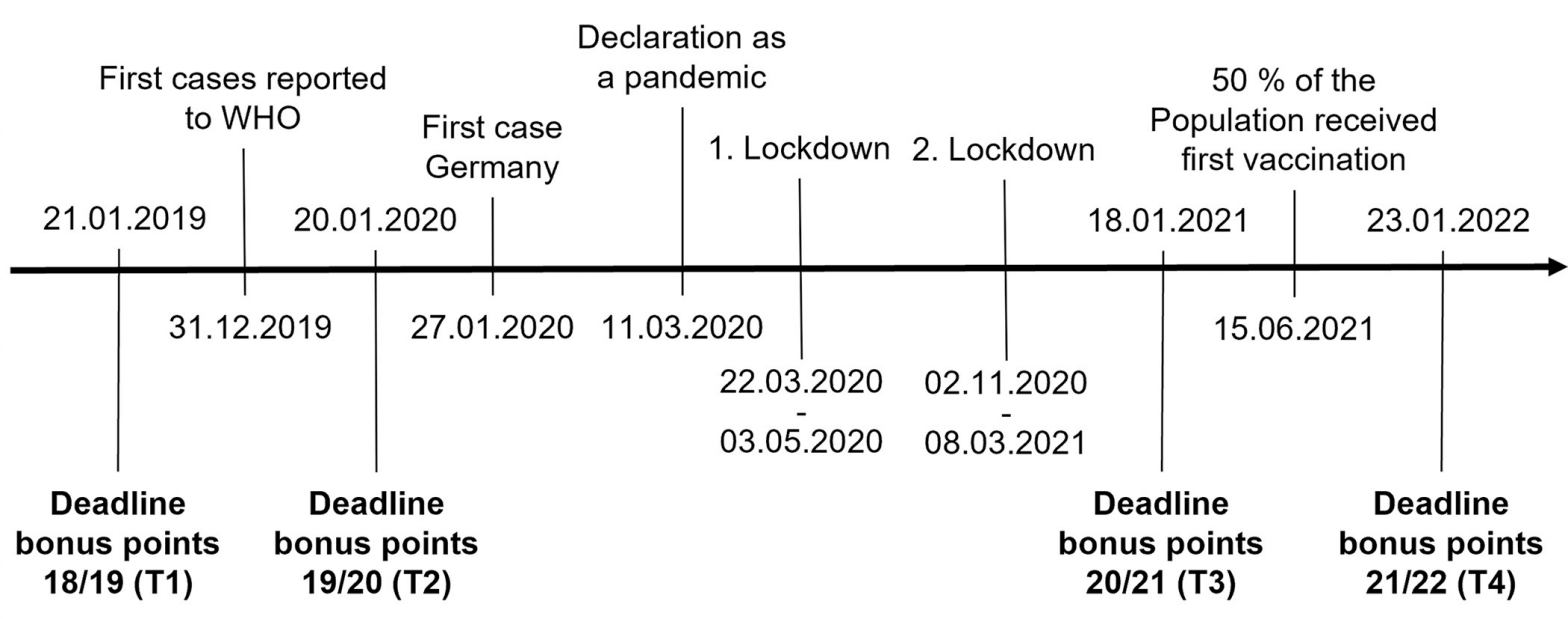
Measurement time point (T) of the study and important COVID-19 events in Germany.

Our study was designed as a longer-term cross-sectional study with four measurement points. The measurement points as well as the most important events regarding the COVID-19 pandemic in Germany [64], are shown in Fig 1.

The first measurement point (T1) was in January 2019, nearly a year before COVID-19 became known. The first disease cases were reported to the WHO on December 31, 2019 [65]. Measurement point two (T2) in January 2020 was a few days before the first case in Germany occurred (January 24, 2020), where the population could not yet guess what would happen a few weeks later. Only then did the COVID-19 cases rapidly increase worldwide and in Germany. Therefore, the WHO declared the disease a pandemic on March 11, 2020 [65]. To keep infection numbers down and to better control the situation, the federal government imposed a strict lockdown on March 22, 2020 [66], severely restricting people’s freedom for seven weeks. A second lockdown followed in December 2020 [64], during which the third measurement point (T3) in January 2020 falls and to which our hypotheses refer. Due to the ongoing restrictions since the beginning of the pandemic, we expect that changes in the values ​​can be observed well at this point in time. In the meantime, a vaccination campaign was already carried out in Germany, whereby the younger population (excluding risk groups), i.e., also the students considered by us, could only be vaccinated with the lifting of the vaccination prioritization from June 2021 onwards [67]. At the fourth measurement point (T4) in January 2022, 61.5 million people in Germany were already vaccinated [68], and compared to time 3, there were significantly fewer restrictions due to the pandemic by the federal government.

The participants in the study were predominantly undergraduate students in the first semesters of their studies. A total of 1,328 people took part in the study. Due to the gender distribution in the study programs, there are significantly more male subjects than female subjects in all data sets. The details are shown in Table 2 for each measurement time point.

**Table 2:** Demographic data of the participants.

Measurement point	T1: WS 18/19	T2: WS 19/20	T3: WS 20/21	T4: WS 21/22
Number of subjects	346	383	285	314
Gender (male/female)	74% / 26%	74% / 26%	71% / 29%	66% / 34%
Degree course (B.Sc. / M.Sc.)	93% / 7%	88% / 12%	94% / 6%	94% / 6%

### Data collection

The approach of value-focused decision-making is the basis for the data collected in this study. As explained before, the participants reflect on their personal values in a career decision currently relevant to them, which they elaborate in a structured manner in the Entscheidungsnavi (S4 Fig). The students were able to reflect on 12 basic values on a continuous scale between "rather less important" (corresponds to value 0) and "particularly important" (corresponds to value 80).

In addition to the values, we also used five questions in 2021 and 2022 to record how much students were affected financially, health-wise, and socially by the Corona pandemic (Table 3).

**Table 3:** Additional questions on being affected by the Corona pandemic.

**To what extent can you agree with the following statements?**
(0 = strongly worsened; 1 = worsened; 2 = slightly worsened; 3 = no change; 4 = slightly improved; 5 = improved; 6 = strongly improved; additionally "no statement" possible)
How has your financial situation changed as a result of the Corona pandemic?
How has your health situation changed as a result of the Corona pandemic?
How has your social life changed as a result of the Corona pandemic?
How has your family/friends’ financial situation changed due to the Corona pandemic?
How has your family/friends’ health situation changed due to the Corona pandemic?

### Analysis

The analysis of the data obtained by the Entscheidungsnavi was carried out step by step. In the first step, all decision projects processed by the Entscheidungsnavi were checked for completeness and extensive elaboration by the chair’s staff. Incomplete and not seriously edited data sets were sorted out. In the second step, the participants’ value priorities were normalized. This study aims to determine the value priorities, i.e., the relative importance between values. In principle, individuals may interpret the endpoint-named scale ("small impact" to "high impact") of the importance of values in the Entscheidungsnavi differently. For such scales, some people use them only in the upper range, others in the lower range, but only a few uses the whole range. Individual differences must therefore be excluded to achieve high accuracy in the measurement. For this purpose, we use the procedure of Schwartz [36], who proposes subtracting the average value of the individual value weights from each value for each data set. This procedure converts the value weights of each participant into relative importance values. At the same time, this procedure tests mean stability rather than rank stability. The analysis of mean stability has the advantage that a more differentiated analysis is possible [11]. In the third step, we analyzed the change in value priorities of the four measurement time points using ANOVA. For further detailed analyses to evaluate the established hypotheses, we performed pairwise comparisons in the form of t-tests. To better interpret the data, we considered the effect sizes according to Cohen [69], which allows an understanding independent of the sample size. Here, we refer to Gignac and Szodorai’s [70] recommendations for the classification of effect sizes. They recommend effect sizes of 0.10 as relatively small, 0.20 as typical, and effect sizes of 0.30 and above as relatively large.

## Results

We explain the results in the following three sub-sections. In the first sub-section, we look at how the coronavirus pandemic affected people. In the second sub-section, we describe the differences between the four measurement points. In the following sub-section, we test the hypotheses stated at the beginning. In the fourth sub-section, we analyze the data for possible gender effects. In the last sub-section, we discuss the results and present the first indications of the influence of the value system on the participants’ objective systems.

### Corona pandemic affectedness

In 2021 and 2022, we explicitly asked students how much their financial, health, and social situations had changed due to the coronavirus pandemic. The results are shown in Table 4.

**Table 4:** Mean values, standard deviation (SD) and significance levels (Mann-Whitney test) of the responses to the additional questions (0 = strongly worsened, 3 = no change, 6 = strongly improved).

Impact of the pandemic	T3: January 2021		T4: January 2022		Mann-Whitney
	Mean	SD	Mean	SD	Sig. (2-sided)
Financial	3.02	1.07	3.06	1.25	.*892*
Health	2.52	1.17	2.19	1.16	*<* .*001*
Social	0.83	0.97	1.33	1.13	*<* .*001*
Family/friends financial	2.53	1.02	2.70	1.07	.*088*
Family/friends health	2.27	1.00	2.15	0.93	.*099*

Overall, students in both years indicate that their financial situation has not worsened, and there is no significant deviation from the value 3 (= no change) (Mann-Whitney Asympt. Sig. 2-sided *p =* .*403* and *p =* .*777*). This supports our expectation that the priority of the value *Financial security* should not have changed significantly. For all other questions regarding their private situation, there is a significant deviation from the value three and thus a change due to the coronavirus pandemic (Mann-Whitney asympt. Sig. 2-sided *p <* .*001*). On average, respondents describe the financial situation of family and friends as only slightly worsened. Students also rate their health situation as only slightly worsened, as does the situation of family/friends. In contrast, they perceive the restrictions in their social life as a significant deterioration.

Between 2021 and 2022, significant mean differences are shown in the description of own health and social limitations. Health limitations are significantly higher in 2022 than in 2021 because more students had COVID-19 than in the previous year. Negative social life restrictions are perceived as considerably higher in 2021 than in 2022 since there were stronger restrictions in January 2021 than in 2022 due to the second lockdown. These results undermine our presumed trends of value priorities in the hypotheses development. We assumed they would mainly be affected by restricted social contact and that financial concerns were irrelevant to them.

### Difference between the measurement times

In the first sub-sections, we illustrated that changes in value priorities could arise either through regular questioning or priming of values or through extreme events. The Corona pandemic can be seen as triggering the questioning of value priorities. Accordingly, significant changes in values are not expected until 2021. Table 5 shows the value priorities’ mean values and standard deviations over the measurement periods. After ANOVA, there are significant mean differences for the values *Family and Partner*, *Environment and Nature*, *Intellectual Fulfillment*, *Freedom and Independence*, and *Power and Leadership*.

**Table 5:** Means (M), standard deviations (SD) and significant levels (η2) of relative value priorities at the four measurement points (T1 -T4).

Value	Measurement-Points				F	η^2^
	T1 M (*SD*)	T2 M (*SD*)	T3 M (*SD*)	T4 M (*SD*)		
**Family and Partner**	10.91 (*14*.*01*)	12.72 (*13*.*28*)	7.82 (*16*.*56*)	10.31 (*14*.*23*)	6.34	.*000*
Friends and Social Relations	8.75 (*12*.*02*)	8.87 (*11*.*44*)	9.39 (*13*.*10*)	9.62 (*12*.*30*)	0.38	.*771*
Justice and Fairness	-2.43 *(12*.*12)*	-1.33 *(13*.*27)*	-1.58 *(12*.*72)*	-2.29 *(12*.*63)*	0.61	.*609*
**Environment and Nature**	-17.12 (*16*.*19*)	-14.97 (*15*.*81*)	-11.83 (*15*.*48*)	-17.28 (*14*.*10*)	8.07	.*000*
**Intellectual Fulfillment**	4.26 (*12*.*82*)	2.42 (*12*.*55*)	5.30 (*13*.*50*)	3.84 (*12*.*44*)	2.99	.*033*
**Freedom and Independence**	4.77 (*14*.*03*)	4.33 (*12*.*75*)	6.39 (*12*.*95*)	7.04 (*12*.*91*)	3.52	.*015*
Excitement and New Experiences	-1.42 *(13*.*81)*	-1.43 *(13*.*50)*	0.35 *(14*.*75)*	0.03 *(13*.*98)*	1.46	.*224*
Competence	1.54 *(11*.*17)*	0.48 *(12*.*11)*	1.34 *(12*.*64)*	0.78 *(11*.*33)*	2.11	.*098*
**Power and Leadership**	-8.69 *(15*.*21)*	-9.96 *(15*.*38)*	-12.21 *(14*.*96)*	-10.55 *(15*.*13)*	2.84	.*037*
Wealth	-12.40 *(17*.*40)*	-12.73 *(16*.*24)*	-15.00 *(16*.*05)*	-12.93 *(15*.*45)*	1.55	.*200*
Financial Security	9.95 (*12*.*11*)	10.20 (*12*.*16*)	9.06 (*13*.*47*)	10.45 (*12*.*40*)	0.70	.*554*
Honesty and Ethics	1.88 *(13*.*25)*	2.36 *(12*.*89)*	0.96 *(12*.*80)*	1.00 *(12*.*66)*	0.94	.*421*

**Fig 2:**
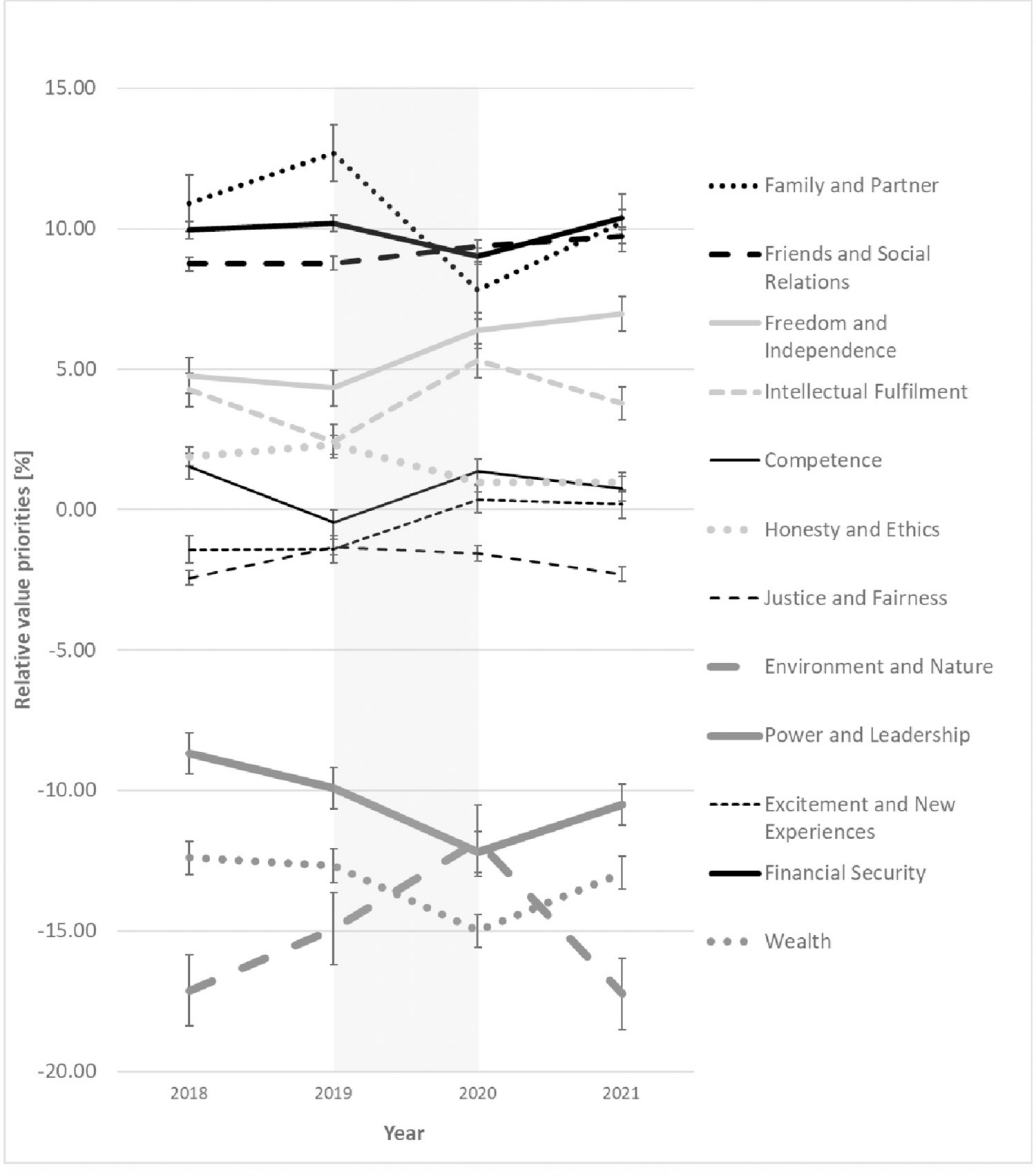
Changes in value priorities over time including standard error bars.

If we look at the mean value trends, the picture shown in Fig 2 emerges. Values prioritized higher (relative value prioritization usually > 0) are shown in black. Values prioritized lower (relative value prioritization usually < 0) are shown in dark gray. Consequently, the values *Family and Partner*, *Financial Security*, *Friends and Social Relations*, *Freedom and Independence*, and *Intellectual Fulfillment* are greatly important to students. In contrast, the values *Power and Leadership*, *Wealth*, and *Environment and Nature* were weighted below average by the respondents. What is interesting for our study is the development of the individual relative value priorities over time, i.e., whether and to what extent changes in direction can be observed by 2021.

We categorize the changes in value priorities over time into three groups. The first group is value changes without statistical significance, the second group is value changes with statistical significance and bounce-back effect, and the third group is longer-term value changes.

The first group (thin lines) includes the values *Friends and Social Relations*, *Justice and Fairness*, *Excitement and New Experiences*, *Competence*, *Wealth*, *Financial Security* and *Honesty and Ethics*. For these values, the ANOVA showed no significant changes (*p >* .*050*). As shown in the last sub-section, these values are not particularly affected by the Corona pandemic; accordingly, we did not expect a change. The exception is the value *Friends and Social Relations*. The value trend is examined in more detail in the hypothesis verification in the next sub-section.

The second category of values (thick, dotted/dashed lines) comprises the values *Family and Partner*, *Environment and Nature*, *Intellectual Fulfillment*, and, to a somewhat lesser extent, *Power and Leadership*. For all values, there is a significant change in value by ANOVA and t-test between 2020 and 2021 (*p <* .*050*). However, after 2021, the value changes moved back toward pre-crisis levels in 2022. This effect is known in the literature as the bounce-back effect [47]. The exact courses, including the p-values and effect sizes, are presented in the next sub-section.

The third category (thick black line) comprises the value *Freedom and Independence*. This is the only value for which a significant change in value priority is maintained in 2022. Since the WHO has not yet declared the pandemic over at the last time of our measurement, it is not yet possible to speak of a long-term effect, even though this value is a possible candidate for it.

### Hypotheses testing

To verify the hypotheses, in the following, we test the mean differences between the measurement time points for statistical significance and evaluate them with the help of the effect sizes.

**Fig 3:**
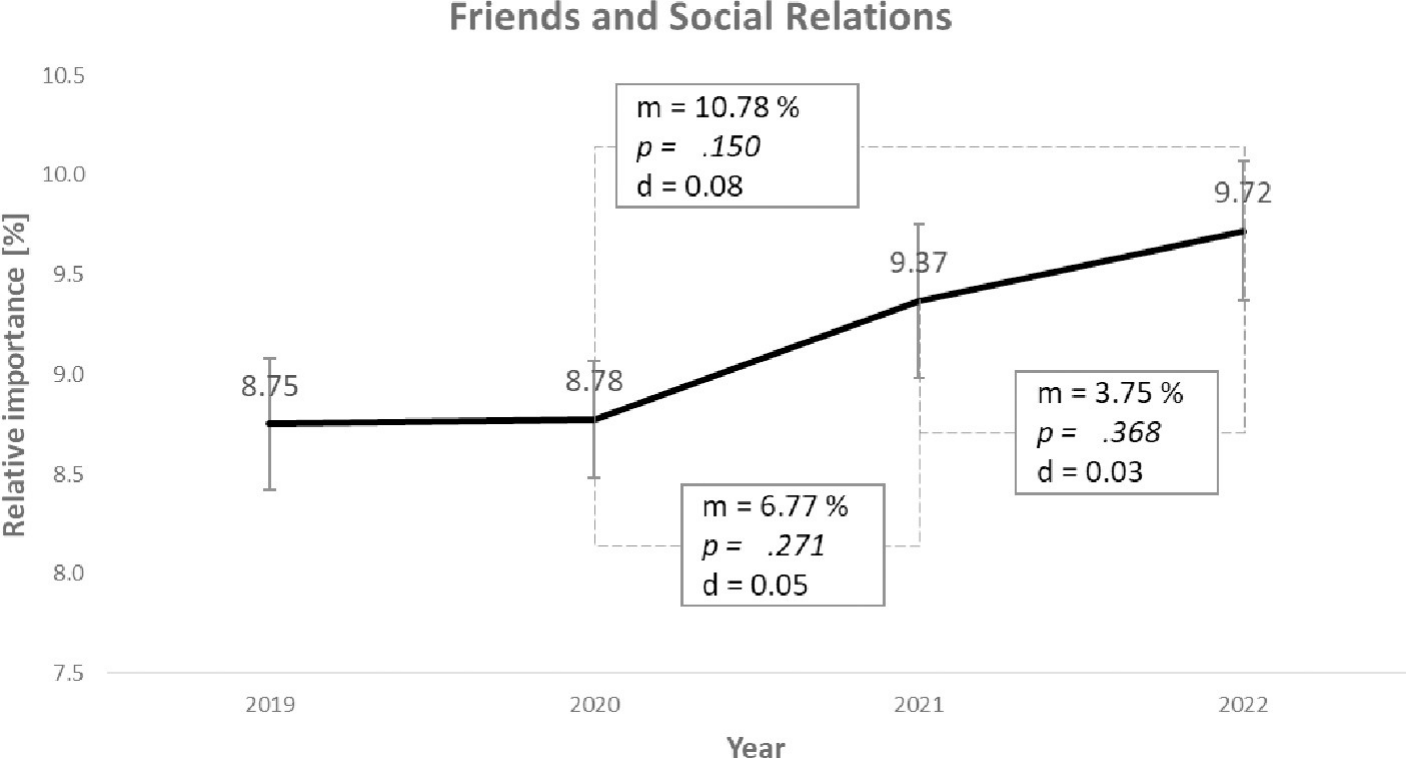
Course of the average value priorities (m) of the *Friends and Social Relations* value with significance levels (p), effect sizes (d) and standard error bars.

The *Friends and Social Relations* value falls into Group 1 of non-significant changes. However, we hypothesized that this would increase during the pandemic. Fig 3 shows the course of the average value priorities over the respective measurement points.

In 2019 (T1), this value had an average importance of 8.75 and increased to 8.78 in 2020 (T2). In 2021 (T3), the value priority further increased to 9.37, and in 2022 (T4) to 9.72. Significance levels for the changes are *p =* .*489* and *p =* .*271*, and *p =* .*368*. The effect sizes are d = 0.00, d = 0.05, and d = 0.03, respectively, and they turn out to be minimal. When the second measurement time point is compared to the third, the average value priority increases by 0.95 (*p =* .*150* and d = 0.08). Overall, although there is an increase according to our hypothesis, it is minimal and therefore not significant. Hypothesis 1 must therefore be rejected.

**Fig 4:**
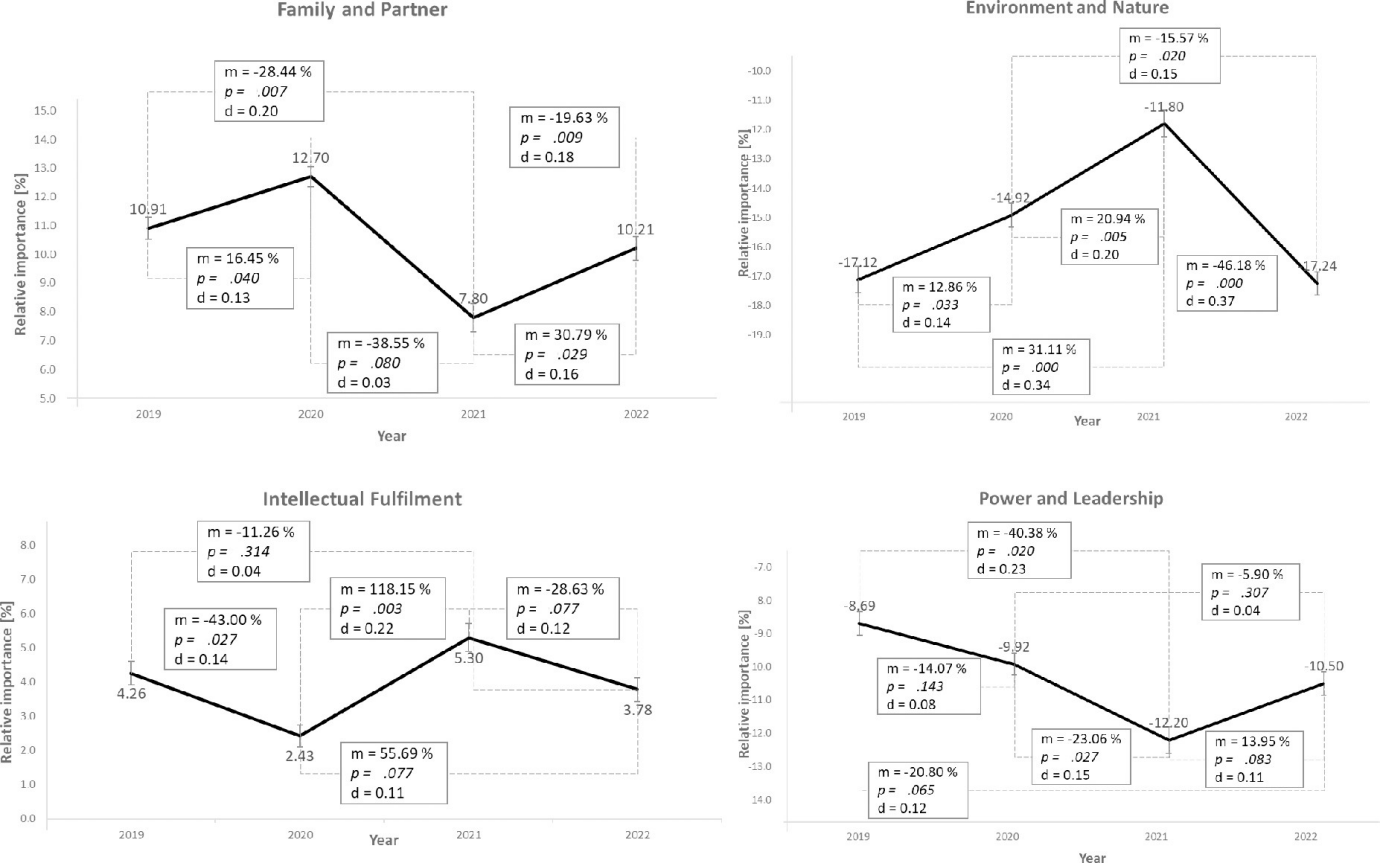
Course of the average value priorities (m) of *Family and Partner*, *Environment and Nature*, *Intellectual Fulfillment*, and *Power and Leadership* with significance levels (p), effect sizes (d) and standard error bars.

The values *Family and Partner*, *Environment and Nature*, *Intellectual Fulfillment*, and *Power and Leadership* belong to group 2, the significant changes in value priorities with a bounce-back effect. Fig 4 shows their courses.

In 2019 (T1), the Family and Partner value had an average relative importance of 10.91 and increased to 12.70 in 2020 (T2). Subsequently, the value priority decreased to 7.80 in 2021 (T3) and again increased to 10.21 in 2022 (T4), with significance levels for the changes all less than 0.1 (*p =* .*040*, *p =* .*000*, *and p =* .*029*). Hypothesis 2, "Family and Partner value priority decreases during the pandemic", can thus be confirmed. The effect sizes are d = 0.13, d = 0.33, and d = 0.16 and can be rated as typical to large, according to Gignac and Szodorai [70]. When the first measurement time point is compared to the third, the average value priority decreases by 28.51% (*p =* .*007*) with a typical effect size (d = 0.20). Overall, the coronavirus pandemic thus significantly decreased the average value priority. The magnitude of the effect size also suggests that the pandemic mitigation measures strongly affect the average value priority here. At T4, there is already a bounce-back effect and, thus, a trend reversal. However, the pre-crisis level has not yet been reached.

In 2019 (T1), the value *Environment and Nature* had an average relative importance of -17.12 in the students’ value systems. The negative sign here means that students prioritize this value at a lower-than-average level than other values. This value priority increased to -14.92 in 2020 (T2) and further to -11.80 in 2021 (T3). Subsequently, the relative importance decreased to -17.24 by 2022 (T4), with significance levels for the changes being *p =* .*033*, *p =* .*005*, and *p =* .*000*. The effect sizes are d = 0.14, d = 0.20, and d = 0.37. In T3, the value priority increased significantly compared to the two previous measurement points (*p =* .*000* for 2019 and *p =* .*005* for 2020), so hypothesis 3, "The value priority of *Environment and Nature* increases during the pandemic", can be confirmed. At the same time, the bounce-back effect with an effect strength of d = 0.37, which can be rated as large, is remarkable.

In 2019 (T1), the value *Intellectual Fulfillment* possessed an average relative importance of 4.26 in students’ value systems. This value priority dropped to 2.43 in 2020 (T2), rose to 5.30 by 2021 (T3), and then dropped to 3.78 in 2022 (T4). Thus, this value gained relative importance in the coronavirus pandemic, although a subsequent bounce-back effect is also observed. Here, the significance levels for the changes are *p =* .*027*, *p =* .*003*, and *p =* .*009*. The effect sizes are d = 0.14, d = 0.22, and d = 0.18. Overall, Hypothesis 4b, "The value priority of *Intellectual Fulfillment* increases during the pandemic", can thus be accepted with a typical effect size.

In addition to the values addressed in the hypotheses, there was a further value with significant changes in value priorities. In 2019 (T1), the value *Power and Leadership* had an average relative importance of -8.69 in the students’ value systems. This value priority decreased to -9.92 in 2020 (T2) and -12.20 in 2021 (T3) before increasing to -10.50 in 2022 (T4). Here, the significance levels for the changes are *p =* .*143*, *p =* .*027*, and *p =* .*083*. The effect sizes are d = 0.08, d = 0.15, and d = 0.04. Thus, the change from T2 to T3 is significant at *p <* .*050*, and a bounce-back effect follows again. One explanation for why the students rated this value lower in T3 may be that the possibilities regarding power and leadership were perceived as limited due to the imposed lockdown.

**Fig 5:**
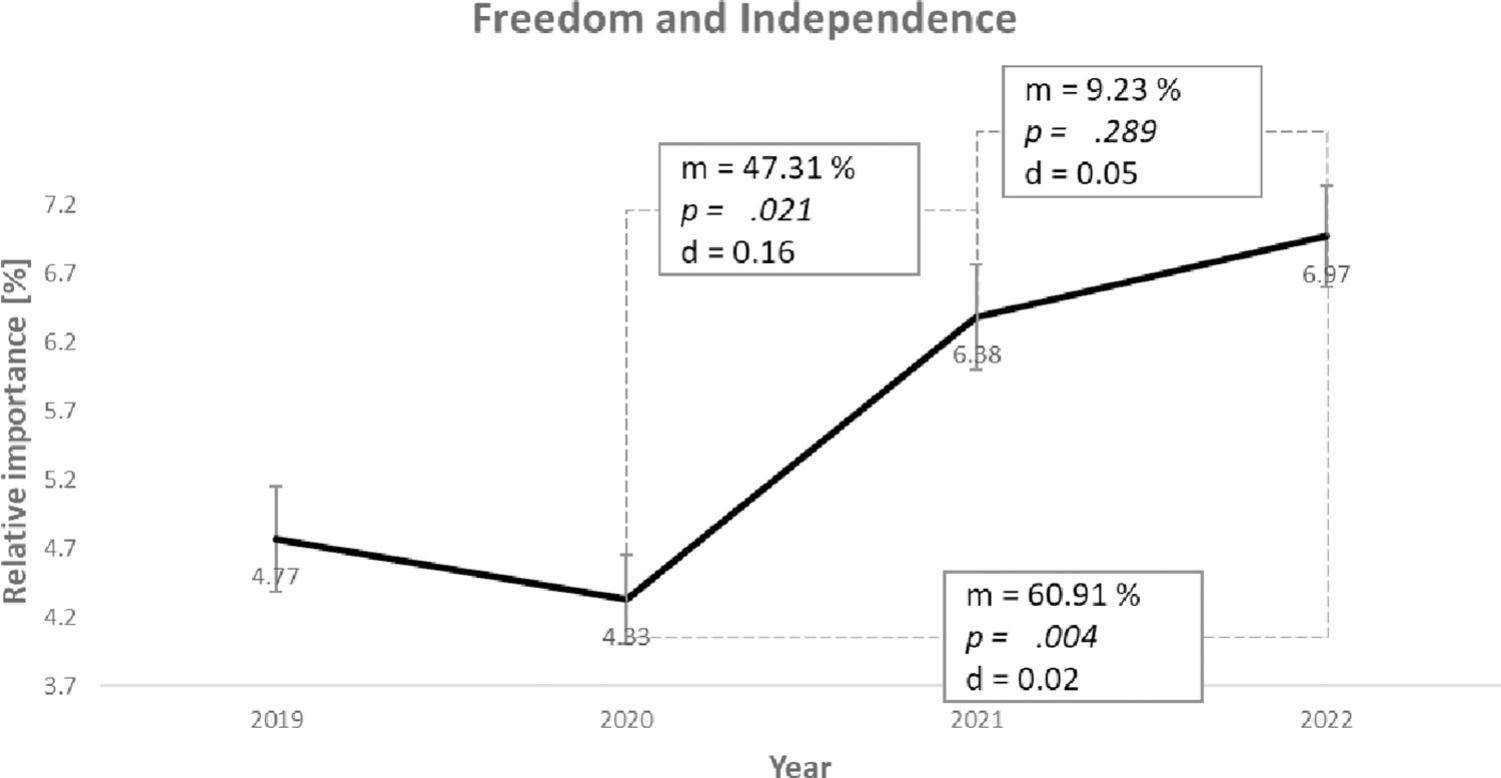
Course of the average value priorities (m) of the value *Freedom and Independence* with significance levels (p), effect sizes (d) and standard error bars.

*Freedom and Independence* belongs to group 3, characterized by a significant value change without a subsequent bounce-back effect. Fig 5 shows the course of the average value priorities over the respective measurement times.

In 2019 (T1), this value had an average importance of 4.77 in the students’ value systems. This value priority decreased slightly to 4.33 in 2020 (T2) and increased to 6.38 (T3) and 6.97 (T4) in the following years, with significance levels *p =* .*329*, *p =* .*0201*, and *p =* .*289*. The effect sizes are d = 0.03, d = 0.16, and d = 0.05, respectively. Thus, the change from T2 to T3 is significant at *p <* .*050* with small to moderate effect sizes so that hypothesis 4a can be confirmed. When T2 is compared to T4, the average value priority increased significantly by 2.64 (*p =* .*004*) and an effect size of d = 0.21, which is medium size according to Gignac and Szodorai [70]. Overall, it can be said that the value priority has increased significantly. The lack of a bounce-back effect may mean that this value priority will stay higher in the long term. However, it is equally possible that the bounce-back effect will only occur with a delay.

### Gender differences

In addition to verifying the hypotheses, we analyzed whether gender effects are evident. Bonjanowska et al. report that both men and women are negatively affected by COVID-19. However, women showed a stronger negative affect than men on well-being [13]. We examined the corona pandemic affectedness at the beginning of the section and now differentiate this analysis according to gender (Table 6).

**Table 6:** Mean values, standard deviation (SD) and significance levels (Mann-Whitney test) of the responses to the additional questions (0 = strongly worsened, 3 = no change, 6 = strongly improved) divided by gender.

Impact of the pandemic	Female		Male		Mann-Whitney
	Mean	SD	Mean	SD	Sig. (2-sided)
Financial	3.84	1.04	4.06	1.14	.*104*
Health	3.43	1.18	3.56	1.21	.*376*
Social	1.79	0.98	1.82	0.96	.*795*
Family/friends financial	3.33	1.14	3.62	1.02	.*035*
Family/friends health	3.18	0.97	3.29	1.04	.*368*

The financial, health and social situation influences well-being. Our data show a deterioration only for the impact on the social situation. Therefore, our results do not directly support the findings of Bonjanowska et al. [13]. Nevertheless, a similar trend can be observed: On average, the information given by women is below that of men, which means that they perceive the impact of the pandemic to be greater. Regarding the financial situations of participants’ families, this effect is significant: Male subjects rate the financial situation of family/friends (mean value 3.63) significantly (*p =* .*035*) more positively than women (mean value 3.33).

**Fig 6:**
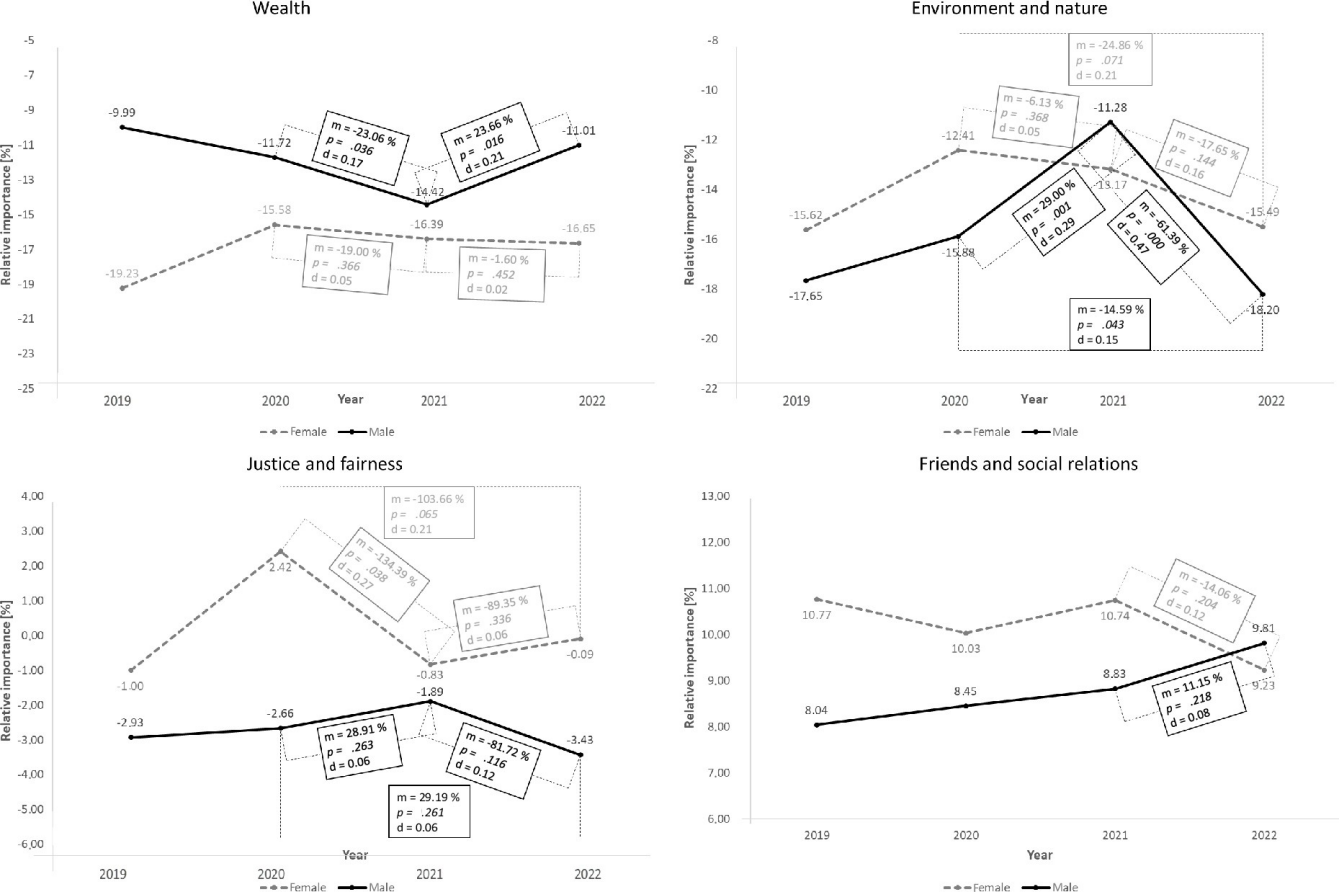
Gender differences in value priorities with the average value priorities (m), significance levels (p), effect sizes (d) and standard error bars for values *Wealth*, *Environment and nature*, *Justice and fairness* and *Friends and social relations*.

On top of that, we analyzed if there were differences in value priority courses between the genders. Fig 6 shows the courses of value priorities for significant differences between genders. We did not find significant differences in the value trajectories for the other values due to the coronavirus pandemic (S5 Fig).

Overall, there are hardly any differences in the trends of value priorities between the genders. Exceptions are the trends for the values Wealth, Environment and Nature and Justice and Fairness. Looking at Wealth’s value trend (Fig 6, top left), we see that the value priority drops significantly between T2 and T3 only for the male subjects and then rises again (significantly). For the female subjects, the value of T4 is even lower than that of T2, although not significantly. The difference between the sexes is visible in the Environment and Nature value curve (Fig 6, top right). While the results for the male subjects are consistent with the overall trend, the trend for the female subjects shows a steady downward movement from 2020 to 2022. We measured no increase in value priority for the female students surveyed during the coronavirus pandemic. The value progression of *Justice and Fairness* is different for the two genders (Fig 6, bottom left). We can see that the female subjects reacted much more strongly to the Corona pandemic, and the value priority dropped significantly (*p =* .*038*) for them and did not increase significantly afterward. Overall, the value priority is lower than the 2020 level. In contrast, we see no significant changes in the value priority in the male subjects. However, the difference may also be due to chance because of the relatively small sample size of women. For the value *Friends and Social Relations* the graphs are congruent. Only in 2022 the priority drops for the male subjects and increases for the female subjects. However, both changes are not significant.

The slight differences in value priority changes between genders are consistent with other researchers’ findings [36]. Differences can be seen between the sexes, but these differences are smaller than differences attributed to age or particular life experiences [11].

## Discussion

In this paper, we examined the impact of the coronavirus pandemic on the value priorities of German students. For data collection, we used the decision support tool Entscheidungsnavi and analyzed value priorities at two points in time before the COVID-19 outbreak and at two points afterward. We found that most value-priority changes are not sustained but are subject to a bounce-back effect. We found this to be the case for four values (*Friends and Social Relations*, *Environment and Nature*, *Intellectual Fulfillment*, and *Power and Leadership*). They showed a significant change at the third point in time (T3, after the outbreak of the Corona virus) and a return to the original level at the fourth point in time (T4). Only in the case of the *Freedom and Independence* value, there is a difference between T1 and T4 that is still significant (*p <* .*050*). Nevertheless, a retardation of the effect at the pandemic onset can be seen for this value (Fig 5), and it is possible that this effect is not yet complete. Bounce-back effects have also occurred following other extreme events (e.g., [13, 14, 47]) and are attributed to the adaptive capacity of humans. At the first moment, people are overwhelmed by the extreme event. Still, they can quickly adapt to the new situation, and their initial value changes return to the pre-crisis level sooner or later. This behavior can be explained by Bardi and Godwin´s model of value change [9]. As the situation in Germany went to a pre-pandemic level, the environmental cues that initiated the change in value priorities fell away, and the initial change did not trigger a schema change of the people. The only exception may be the value of *Freedom and Independence*. We found a significant increase in the value *Freedom and Independence* without a previous return to the pre-crisis level. Here, a further but decelerated increase was evident at the last measurement point (T4), so a bounce-back effect may not occur until later or may not occur at all.

In analyzing the trends caused by the coronavirus pandemic, we are supported by the measurement data from 2019. The fact that not just one but two measurement points were already available before the pandemic means that general trends can also be detected, and the influence of the pandemic can be better put into context. For the *Family and Partner* value, we could discover a positive trend before the pandemic onset that changed to the opposite in T3 (*p <* .*010*). Consistent with our results (Fig 2), Family values are known to be very high in Germany, and for young adults the relationship with their parents shows an increasing trend between 2002 and 2019 [71, 72]. The trend changes in T3 after the pandemic onset that can be explained by a temporary over-fulfillment of this need and the subsequent bounce-back effect in T4 are another indication of the (strong) effect of the coronavirus pandemic on this value priority. We expect that in the future, the *Family and Partner* value will further increase and re-adjust to its previous level. The *Intellectual Fulfillment* value shows a significant decrease between 2019 and 2020 that reversed after the pandemic onset. Even after an extensive search, we could not find a single event to explain that trend. We could only guess that sparse eligibility of participation [73] and insufficient transformation in times of increasing digitalization worldwide may be one aspect of this observation. For the value weights of *Environment and Nature*, we already discovered an upward trend between 2019 and 2020. One reason may be the Fridays for future movement, which was initiated by Greta Thunberg in August 2018 and supported by young people in Germany since January 2019 [74]. The prevailing trend was enhanced after the COVID-19 outbreak and continued to increase for the moment. Especially the first lockdown caused people in Germany to spend much more time in nature. However, at the fourth measurement time point, the value priority dropped to the 2019 level. This can be the result of an over-fulfillment of needs as well as an effect of reduced climate reporting. Due to the Corona pandemic, topics such as climate change and protecting nature and the environment took a back seat, and issues such as vaccinations and personal protection were addressed more intensively [75, 76].

We wanted to investigate the influence of the corona pandemic on value priorities. Research to date only looked into immediate effects after the first lockdown [13] or in countries with a “mild pandemic” [14]. Therefore, we analyzed the situation in Germany, as there have been stricter conditions. We used a different approach measuring the change of value priorities. Instead of asking subjects to fill out a questionnaire, we asked them to indicate their value priorities while working on an important real-life decision. In order to better compare our results with previous findings, we used the classification of Table 1, which lists our values and each corresponding value in Schwartz’s value system. We found similar behavior in the subjects (see following paragraphs) even though we used a different measurement approach. This shows us that both approaches seem to be reasonable to use when measuring changes in value priorities.

Bojanowska et al. [13] examined how personal values of Polish adults changed immediately, i.e., two and four weeks after the first lockdown. They reported an increase in the values self-direction (thought), conformity (rules), humility and universalism (nature and tolerance) an both points in time. Regarding the value change in our study in T3, our results show similarities for the values *Intellectual Fulfillment* which is reflected by self-direction (thought), and *Environment and Nature*, which is part of the universalism category. For the values security, interpersonal conformity, benevolence caring and universalism concern, Bojanowska et al. reported an increase two weeks into the lockdown that was followed by a bounce-back effect so that these increases already diminished four weeks into the lockdown. In accordance with their findings we found no significant changes for the values *Financial Security* and *Honesty and Ethics*. The decrease in the *Family and Partner* value that we found seems not to be an instant reaction to the lockdown but a consequence of the associated limitations as we stated above. The same may be the reason for the values *Freedom and Independence* and the *Power and Leadership* where Bojanowska et al. reported no significant changes in contrast to our results.

By comparing our results with the findings of Daniel et al. [14], we have in mind that the first wave in March/April did occur in both countries at the same time, whereas the second wave already hit Australia from July to September 2020 but Germany not until November 2020 and that our first measurement point after the pandemic’s start is posterior to their survey. Daniel et al. report a decrease in universalism values early into the pandemic onset (April 2020), which persisted and was followed by a decrease in benevolence values (measurement point in November/December 2020). These observations are similar to our findings with a decrease in the values *Family and Partner* and *Environment and Nature* in T3. Since we have one further observation point T4 almost 2 years into the pandemic onset, our results also suggest that this decrease is temporary and followed by a later bounce-back effect. Daniel et al. furthermore report a value change in the openness to change dimension with different change patterns within this dimension. Their results show a decreased value importance at pandemic onset that emanated from a decrease in stimulation values and then a bounce-back effect stemming mainly from an increase in self-direction values. The latter is similar to the ascending trend in our corresponding value *Freedom and Independence*. For the self-enhancement dimension, the results of Daniel et al. suggest no significant changes, whereas our results imply a temporary decrease. One explanation may be country-specific differences or the different measurement methods. Here, further research is necessary.

To sum it up, we can note the following regarding the higher-level dimensions of Schwartz’ value theory: Our results suggest that within the dimension Self-Transcendence, there are opposing tendencies in the way that *Benevolence* values were temporarily prioritized lower due to increased need satisfaction and *Nature* was prioritized higher. In the dimension Openness to Change, we observe a temporarily higher prioritization due to the pandemic, and in the dimension Self-Enhancement, a temporarily lower prioritization.

### Limitations and outlook

This study has some limitations. One limitation is the sample selection. First, the sample is German undergraduate students, so the results are only valid for this group. Second, not the same individuals were interviewed during the pandemic. However, the composition of the respondents was very similar in each of the four years. At the same time, the decision context was identical so that we can assume a largely homogeneous group. Moreover, one argument for not surveying the same students over four years is that students’ value priorities continue to develop over their studies, so this could otherwise have led to biases [29, 77, 78]. Our measurement gives us the advantage that the results do not need to be adjusted for this effect. In future research, the limitations highlighted could be addressed. Thus, changes in value priorities in the overall population of Germany could be studied and related to real decision behavior. Such a study would have two advantages at once. First, the magnitude of the influence of value changes on decision behavior could be determined, and second, such a study could further validate the results analyzed here.

Another limitation is our list of twelve values. Since the Entscheidungsnavi is a practical tool designed to help people make real decisions in a more reflective manner, the twelve values were collected as individual items. In order to minimize measurement errors caused by the participants’ deviating interpretation of the indicators, the items were developed by a small group of experts in brainstorming sessions on the basis of value theory. However, as shown, this also means that our results can only be compared to a limited extent with other studies that are based, for example, on Schwartz’s values. The latter independently poses a problem since no worldwide standard exists here. Likewise, the results of Bojanowska et al. [13] and Daniel et al. [14], which both refer to Schwartz’s values, use different degrees of differentiation (10 values vs. 19) and different outcome methods (portrait of values questionnaire vs. best worst survey). For validity reasons, multi-item latent variable scales are generally preferable. However, there are also studies arguing that the performance of single-item test scales can be even better than that of multi-item scales [79]. To better assess validity, we are planning a validation study using established scores to verify our items. And to obtain further information about reliability, we plan to conduct repeated surveys with the same participants.

## Conclusion

The coronavirus pandemic has had a powerful impact on people’s lives. The world’s governments used and still use, in some cases, strong restrictions on contact and freedom to get the pandemic under control. This exceptional situation is an opportunity for behavioral research. Thus, the influence of extreme situations on the psychology and behavior of people can be studied. We used data collected over four years examining students’ values at a German university. Since human behavior depends on personal values, this scientific paper investigated the pandemic’s influence on German students’ value system.

With our approach, we could generate several insights about the value change of German students: First, values can be seen as a relatively stable construct even in extreme situations. In this context, stability means that value change is only measurable if the people do adapt to the situation, or the situation changes back to its initial level. Second, the change in value priorities can be explained with the model of Bardi and Goodwin [9]. Their approach helps to identify relevant situations for changes in value priorities. Furthermore, their model does not show the direction of the value change but only indicates the reason for the change. Third, the Entscheidungsnavi is another way to measure value change over time.

Looking into the change of value priorities induced by the pandemic, our results suggest that the coronavirus pandemic has a moderate to strong effect on students’ value priorities. We found significant changes associated with the pandemic for five values. Among these, most of the changes in values showed a bounce-back effect. This means that the values return to the original level after a change. The values of *Family and Partner*, and *Power and Leadership* fell with the onset of the pandemic but rose back to pre-pandemic levels as the pandemic progressed. The value priorities of *Intellectual Fulfillment* and *Environment and Nature* increased at the start of the pandemic and fell back to pre-pandemic levels throughout the pandemic. Only the *Freedom and Independence* value shows no bounce-back effect so far. Further research is needed here to determine whether this is a long-term change in value prioritization or whether the bounce-back effect is slower for this value. The research on the influence of gender on value changes comes to the same conclusion as other researchers have already done [11]: There are gender differences, but they are only of a small magnitude.

## Supporting information

S1 Fig Value system according to Schwartz. (TIF)

S2 Fig Simplified model of value change based on Bardi & Godwin. (TIF)

S3 Fig Process steps in the Entscheidungsnavi. (TIF)

S4 Fig Value items. (TIF)

S5 Fig Value priorities over time and by gender with no significant differences. (TIF)

S1 Data (XLSX)

S2 Data (XLSX)

## References

[1] Vindegaard N, Benros ME. COVID-19 pandemic and mental health consequences: Systematic review of the current evidence. Brain Behav Immun 2020;89:531–42. doi: 10.1016/j.bbi.2020.05.048 ; PubMed Central PMCID: PMC7260522. 32485289 PMC7260522

[2] Cifuentes-Faura J. The Importance of Behavioral Economics during COVID-19. JEBS 2020;12(3(J)):70–4.

[3] Pedrosa AL, Bitencourt L, Fróes ACF, Cazumbá MLB, Campos RGB, Brito SBCS de, et al. Emotional, Behavioral, and Psychological Impact of the COVID-19 Pandemic. Front Psychol 2020;11:566212. doi: 10.3389/fpsyg.2020.566212 ; PubMed Central PMCID: PMC7561666. 33117234 PMC7561666

[4] Baddeley M. Hoarding in the age of COVID-19. Journal of Behavioral Economics for Policy 2020;4(COVID-19 Special Issue):69–75.

[5] David J, Visvalingam S, Norberg MM. Why did all the toilet paper disappear? Distinguishing between panic buying and hoarding during COVID-19. Psychiatry Research 2021;303:114062. doi: 10.1016/j.psychres.2021.114062 ; PubMed Central PMCID: PMC8520319. 34175712 PMC8520319

[6] Bourmistrova NW, Solomon T, Braude P, Strawbridge R, Carter B. Long-term effects of COVID-19 on mental health: A systematic review. J Affect Disord 2022;299:118–25. doi: 10.1016/j.jad.2021.11.031 ; PubMed Central PMCID: PMC8758130. 34798148 PMC8758130

[7] Schuster C, Pinkowski L, Fischer D. Intra-Individual Value Change in Adulthood. Zeitschrift für Psychologie 2019;227(1):42–52.

[8] Bardi A, Buchanan KE, Goodwin R, Slabu L, Robinson M. Value stability and change during self-chosen life transitions: self-selection versus socialization effects. Journal of Personality and Social Psychology 2014;106(1):131–47. doi: 10.1037/a0034818 . 24219783

[9] Bardi A, Goodwin R. The Dual Route to Value Change: Individual Processes and Cultural Moderators. Journal of Cross-Cultural Psychology 2011;42(2):271–87.

[10] Allport GW. Pattern and growth in personality. New York: Holt, Rinehart & Winston; 1961. 610 p.

[11] Sagiv L, Schwartz SH. Personal Values Across Cultures. Annu Rev Psychol 2022;73:517–46. doi: 10.1146/annurev-psych-020821-125100 . 34665670

[12] Potocan V, Nedelko Z. How personal values follow the societal lockdown due to COVID-19: Case of business students in Slovenia. Front Psychol 2023;14:987715. doi: 10.3389/fpsyg.2023.987715 ; PubMed Central PMCID: PMC10140780. 37123289 PMC10140780

[13] Bojanowska A, Kaczmarek ŁD, Koscielniak M, Urbańska B. Changes in values and well-being amidst the COVID-19 pandemic in Poland. PLoS One 2021;16(9):e0255491. doi: 10.1371/journal.pone.0255491 ; PubMed Central PMCID: PMC8443041. 34525095 PMC8443041

[14] Daniel E, Bardi A, Fischer R, Benish-Weisman M, Lee JA. Changes in Personal Values in Pandemic Times. Social Psychological and Personality Science 2022;13(2):572–82.

[15] van de Poel I, Wildt T de, van Kooten Pássaro D. COVID-19 and Changing Values. In: Dennis MJ, Ishmaev G, Umbrello S, van Hoven J den, editors. Values for a post-pandemic future. Philosophy of Engineering and Technology. Vol 40. Cham: Springer; 2022. p. 23–58.

[16] Vecchione M. Basic personal values in the midst of the COVID-19 pandemic in Italy: A two-wave longitudinal study. PLOS ONE 2022;17(9):e0274111. doi: 10.1371/journal.pone.0274111 ; PubMed Central PMCID: PMC9462816. 36084064 PMC9462816

[17] Bardi A, Calogero RM, Mullen B. A new archival approach to the study of values and value—behavior relations: validation of the value lexicon. J Appl Psychol 2008;93(3):483–97. doi: 10.1037/0021-9010.93.3.483 . 18457482

[18] Kluckhohn C. Values and value orientations in the theory of action. In: Parsons T, Sils E, editors. Toward a general theory of action. Cambridge, MA: Harvard University Press; 1951. p. 388–433.

[19] Heckhausen H, Heckhausen J. Motivation und Handeln. 4th ed. Berlin: Springer; 2010.

[20] Biernat M. Motives and Values to Achieve: Different Constructs With Different Effects. Journal of Personality 1989;57(1):69–95.

[21] Kehr HM. Integrating Implicit Motives, Explicit Motives, and Perceived Abilities: The Compensatory Model of Work Motivation and Volition. Academy of Management Review 2004;29(3):479–99.

[22] Rohan MJ. A Rose by Any Name? The Values Construct. Pers Soc Psychol Rev 2000;4(3):255–77.

[23] Hutcheon PD. Value Theory: Towards Conceptual Clarification. The British Journal of Sociology 1972;23(2):172–87.

[24] Staehle WH, Conrad P, Sydow J. Management. Eine verhaltenswissenschaftliche Perspektive. 8th ed. München: Vahlen; 1999.

[25] Rokeach M. The nature of human values. New York: Free Press; 1973.

[26] Rokeach M. Understanding Human Values: Simon and Schuster; 2008.

[27] Schwartz SH. Universals in the Content and Structure of Values: Theoretical Advances and Empirical Tests in 20 Countries. In: Zanna Mark P., editor. Advances in Experimental Social Psychology: Academic Press; 1992. p. 1–65.

[28] Friedman B, Kahn PH, JR, Borning A. Value sensitive design and information systems. In: Zhang P, Galletta D, editors. Human-computer interaction in management information systems: Foundations. New York: M. E. Sharpe; 2006. p. 348–72.

[29] Feather NT. Values in education and society. New York: Free Press; 1975.

[30] Maslow AH. Motivation and Personality. New York: Harper; 1954.

[31] Beatty SE, Kahle LR, Homer P, Misra S. Alternative Measurement Approaches to Consumer Values: The List of Values and the Rokeach Value Survey. Psychology and Marketing 1985;2(3):181–200.

[32] Maio GR. Mental Representations of Social Values. In: Zanna MP, editor. Advances in Experimental Social Psychology: Elsevier; 2010. p. 1–43.

[33] Schwartz SH, Cieciuch J. Measuring the Refined Theory of Individual Values in 49 Cultural Groups: Psychometrics of the Revised Portrait Value Questionnaire. Assessment 2021;29(5):1005–1019. doi: 10.1177/1073191121998760 . 33682477 PMC9131418

[34] Schwartz SH. Are There Universal Aspects in the Structure and Contents of Human Values? Journal of Social Issues 1994;50(4):19–45.

[35] Schwartz SH, Bilsky W. Toward a universal psychological structure of human values. Journal of Personality and Social Psychology 1987;53(3):550–62.

[36] Schwartz SH. An Overview of the Schwartz Theory of Basic Values. Online Readings in Psychology and Culture 2012;2(1).

[37] Schwartz SH, Cieciuch J, Vecchione M, Davidov E, Fischer R, Beierlein C, et al. Refining the theory of basic individual values. Journal of Personality and Social Psychology 2012;103(4):663–88. doi: 10.1037/a0029393 . 22823292

[38] Sheldon KM. Positive value change during college: Normative trends and individual differences. Journal of Research in Personality 2005;39(2):209–23.

[39] Verkasalo M, Lönnqvist J-E, Lipsanen J, Helkama K. European norms and equations for a two dimensional presentation of values as measured with Schwartz’s 21-item portrait values questionnaire. Eur. J. Soc. Psychol. 2009;39(5):780–92.

[40] Schwartz SH. Value Orientations: Measurement, Antecedents and Consequences Across Nations. In: Jowell R, Roberts C, Fitzgerald R, Eva G, editors. Measuring attitudes cross-nationality: Lessons from the European Social Survey. p. 169–203.

[41] Pöge A. Stability and change of values during the formative years: Latent state-trait analyses of adolescents in a seven-wave panel study. J Personality 2020;88(2):266–86. doi: 10.1111/jopy.12484 . 31062361

[42] Bardi A, Lee JA, Hofmann-Towfigh N, Soutar G. The structure of intraindividual value change. Journal of Personality and Social Psychology 2009;97(5):913–29. doi: 10.1037/a0016617 . 19857010

[43] Gouveia VV, Vione KC, Milfont TL, Fischer R. Patterns of Value Change During the Life Span: Some Evidence From a Functional Approach to Values. Pers Soc Psychol Bull 2015;41(9):1276–90. doi: 10.1177/0146167215594189 . 26187119

[44] Robinson OC. Values and adult age: findings from two cohorts of the European Social Survey. Eur J Ageing 2012;10(1):11–23. doi: 10.1007/s10433-012-0247-3 ; PubMed Central PMCID: PMC5549227. 28804279 PMC5549227

[45] Lönnqvist J-E, Leikas S, Verkasalo M. Value change in men and women entering parenthood: New mothers’ value priorities shift towards Conservation values. Personality and Individual Differences 2018;120:47–51.

[46] Sortheix FM, Parker PD, Lechner CM, Schwartz SH. Changes in Young Europeans’ Values During the Global Financial Crisis. Social Psychological and Personality Science 2019;10(1):15–25.

[47] Verkasalo M, Goodwin R, Bezmenova I. Values Following a Major Terrorist Incident: Finnish Adolescent and Student Values Before and After September 11, 2001. Journal of Applied Social Psychology 2006;36(1):144–60.

[48] Daniel E, Fortuna K, Thrun SK, Cioban S, Knafo A. Brief report: early adolescents’ value development at war time. J Adolesc 2013;36(4):651–5. doi: 10.1016/j.adolescence.2013.03.009 . 23849659

[49] Bardi A, Schwartz SH. Relations among Sociopolitical Values in Eastern Europe: Effects of the Communist Experience? Political Psychology 1996;17(3):525–49.

[50] Walper S, Reim J, Schunke A, Berngruber A, Alt P. Die Situation Jugendlicher in der Corona-Krise. München [cited 2023 Oct 4]. Available from: https://www.dji.de/fileadmin/user_upload/bibs2021/2021-05-21_Walper%20et%20al_2021_Die%20Situation%20Jugendlicher%20in%20der%20Coronakrise_1205%20%28003%29.pdf.

[51] Reiss S. Multifaceted Nature of Intrinsic Motivation: The Theory of 16 Basic Desires. Review of General Psychology 2004;8(3):179–93.

[52] Reiss S, Havercamp S. The sensitivity theory of motivation: implications for psychopathology. Behaviour Research and Therapy 1996;34(8):621–32. doi: 10.1016/0005-7967(96)00041-1 . 8870288

[53] Morse JW, Gladkikh TM, Hackenburg DM, Gould RK. COVID-19 and human-nature relationships: Vermonters’ activities in nature and associated nonmaterial values during the pandemic. PLOS ONE 2020;15(12):e0243697. doi: 10.1371/journal.pone.0243697 ; PubMed Central PMCID: PMC7732125. 33306716 PMC7732125

[54] Büssing A, Rodrigues Recchia D, Hein R, Dienberg T. Perceived changes of specific attitudes, perceptions and behaviors during the Corona pandemic and their relation to wellbeing. Health and Quality of Life Outcomes 2020;18(1):374. doi: 10.1186/s12955-020-01623-6 ; PubMed Central PMCID: PMC7702679. 33256755 PMC7702679

[55] Hövermann A. Corona-Zweifel, Unzufriedenheit und Verschwörungsmythen: Erkenntnisse aus zwei Wellen der HBS-Erwerbspersonenbefragung 2020 zu Einstellungen zur Pandemie und den politischen Schutzmaßnahmen. Hans-Böckler Stiftung, Wirtschafts- und Sozialwissenschaftliches Institut (WSI). WSI Policy Brief. Düsseldorf; 2020. 48. Available from: http://hdl.handle.net/10419/226226.

[56] Schöberl S, Kieweg P. Veränderungen im Informationsverhalten in der Corona-Krise und ihre Auswirkungen auf die Sichtweisen junger Menschen. kommges 2021;22(1).

[57] Olkowicz J, Jarosik-Michalak A. The Role of Leadership In the COVID-19 Pandemic Crisis. WSB Journal of Business and Finance 2022;56(1):55–63.

[58] Batty M, Deeken E, Volz AH. Wealth Inequality and COVID-19: Evidence from the Distributional Financial Accounts. FEDS Notes 30 Aug. 2021.

[59] Nitzsch R von, Tönsfeuerborn M, Siebert JU. Decision Skill Training with the Entscheidungsnavi. In: de Almeida A.T., Morais D.C., editor. Innovation for Systems Information and Decision: Second. INSID. Lecture Notes in Business Information Processing. Vol 405. [S.l.]: SPRINGER NATURE; 2020. p. 15–30.

[60] Keeney RL. Value-Focused Thinking: A Path To Creative Decision-Making. Cambridge: Harvard University Press; 1992.

[61] Keeney RL. Creativity in MS/OR: Value-Focused Thinking—Creativity Directed toward Decision Making. Interfaces 1993;23(3):62–7.

[62] Siebert J, Keeney RL. Creating More and Better Alternatives for Decisions Using Objectives. Operations Research 2015;63(5):1144–58.

[63] Parnell GS, Hughes DW, Burk RC, Driscoll PJ, Kucik PD, Morales BL, et al. Invited Review-Survey of Value-Focused Thinking: Applications, Research Developments and Areas for Future Research. J. Multi-Crit. Decis. Anal. 2013;20:49–60.

[64] Imöhl S, Ivanov A. Bundesregierung bestellt 80 Millionen Dosen Omikron-Impfstoff bei Biontech: Die Zusammenfassung der aktuellen Lage seit Ausbruch von Covid-19 im Januar 2020. [Internet]; 2021. Available from: https://www.handelsblatt.com/politik/corona-chronik-bundesregierung-bestellt-80-millionen-dosen-omikron-impfstoff-bei-biontech/25584942.html.

[65] World Health Organization. Listings of WHO’s response to COVID-19. https://www.who.int/news/item/29-06-2020-covidtimeline; 2022.

[66] Bundesregierung Deutschland. 22. März 2020: Regeln zum Corona-Virus [Internet]; 2020. Available from: https://www.bundesregierung.de/breg-de/leichte-sprache/22-maerz-2020-regeln-zum-corona-virus-1733310.

[67] RKI. STIKO-Empfehlungen zur COVID-19 Impfung [Internet]; 2022. Available from: https://www.rki.de/DE/Content/Infekt/Impfen/ImpfungenAZ/COVID-19/Impfempfehlung-Zusfassung.html.

[68] Bundesministerium für Gesundheit. impfdashboard [Internet]. Bundesministerium für Gesundheit; 2023. Available from: https://impfdashboard.de/.

[69] Cohen J. Statistical power analysis for the behavioral sciences. 2nd ed. Hillsdale, N.J: Lawrence Erlbaum Associates; 1988.

[70] Gignac GE, Szodorai ET. Effect size guidelines for individual differences researchers. Personality and Individual Differences 2016;102:74–8.

[71] Bundesministerium für Familie Senioren, Frauen und Jugend. Familie heute. Daten. Fakten. Trends: Familienreport 2020. 2nd ed. Berlin; 2021. Available from: https://www.bmfsfj.de/resource/blob/163108/ceb1abd3901f50a0dc484d899881a223/familienreport-2020-familie-heute-daten-fakten-trends-data.pdf.

[72] Deutscher Familienverband. Ist Familie noch wichtig für junge Menschen? [Internet]. Deutscher Familienverband; 2019. Available from: https://www.deutscher-familienverband.de/ist-familie-noch-wichtig-fuer-junge-menschen/.

[73] Deutsche Telekom Stiftung. Schule motiviert nicht ausreichend zum Lernen [Internet]. Deutsche Telekom Stiftung; 2020. Available from: https://bildungsklick.de/schule/detail/allensbach-umfrage-schule-motiviert-nicht-ausreichend-zum-lernen.

[74] Fridays for Future Deutschland. FFF Neuigkeiten [Internet]. Fridays for Future Deutschland. Available from: https://fridaysforfuture.de/neuigkeiten/page/29/.

[75] Welthungerhilfe. Klimawandel im Schatten von Corona [Internet]. Welthungerhilfe [cited 2022 Nov 4]. Available from: https://www.welthungerhilfe.de/corona-spenden/klimawandel-im-schatten-von-corona.

[76] Krug M, Kuhn P, Lauschner L, Mercsak A, Schulte-Zweckel N. Von Umwelt zu Corona: Der Schwerpunktwechsel in der Berichterstattung während der ersten Welle der Coronapandemie in Deutschland. Momentum Quarterly 2021;10(3):130.

[77] Krishnan VR. Impact of MBA Education on Students’ Values: Two Longitudinal Studies. J Bus Ethics 2008;83(2):233–46.

[78] Krosnick JA, Alwin DF. Aging and susceptibility to attitude change. Journal of Personality and Social Psychology 1989;57(3):416–25. doi: 10.1037//0022-3514.57.3.416 . 2778632

[79] Bergkvist L, Rossiter JR. The Predictive Validity of Multiple-Item versus Single-Item Measures of the Same Constructs. Journal of Marketing Research 2007;44(2):175–84.

