# Supplementary figures and images for: Changes in value priorities due to the COVID-19 pandemic—A 4-year cross-sectional study with German students
Source: PLoS One. 2024 Jan 19;19(1):e0297236. doi: 10.1371/journal.pone.0297236 (PMC10798440; doi:10.1371/journal.pone.0297236)

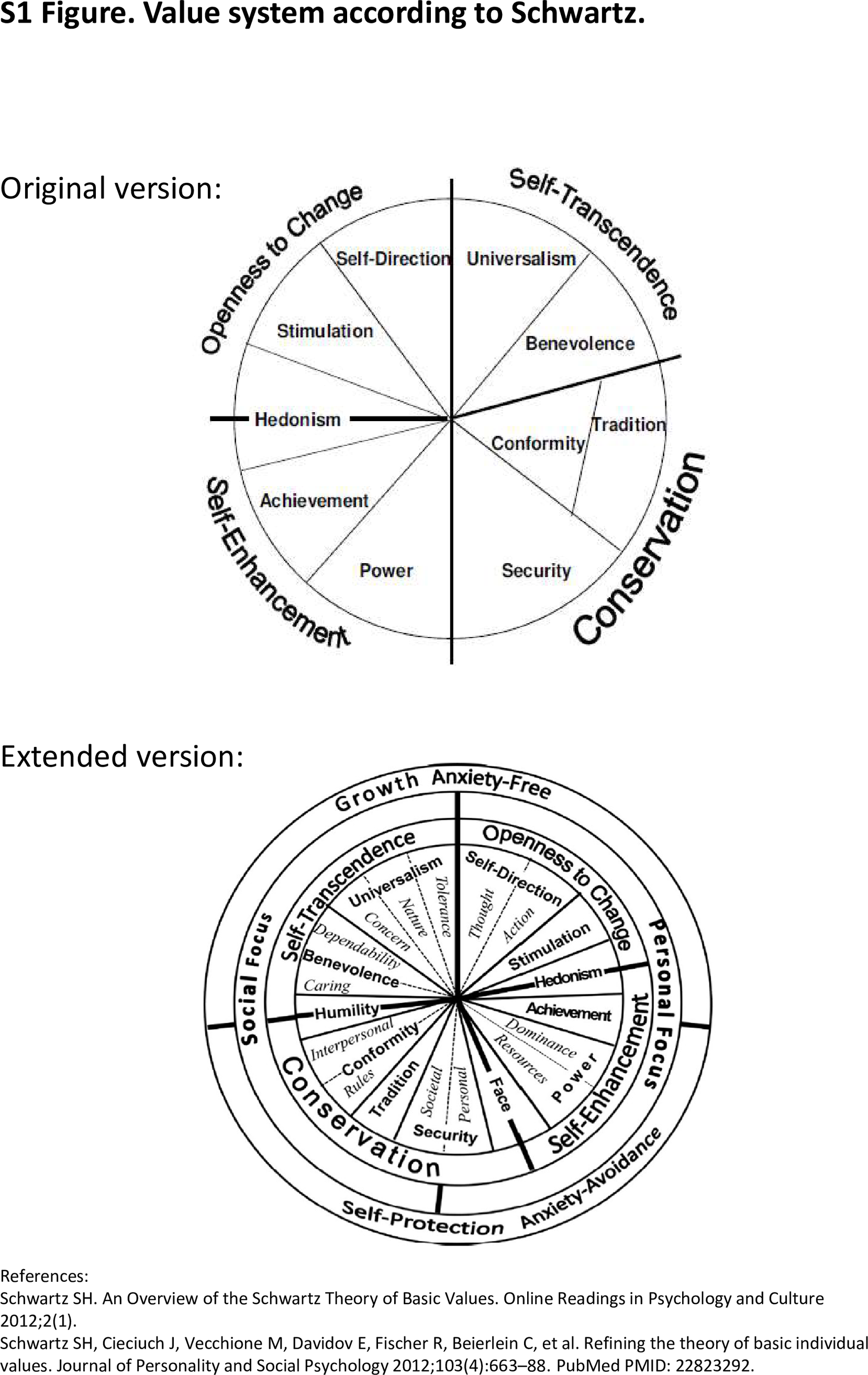

Supplement: S1 Fig — (TIF) [file pone.0297236.s001.tif]

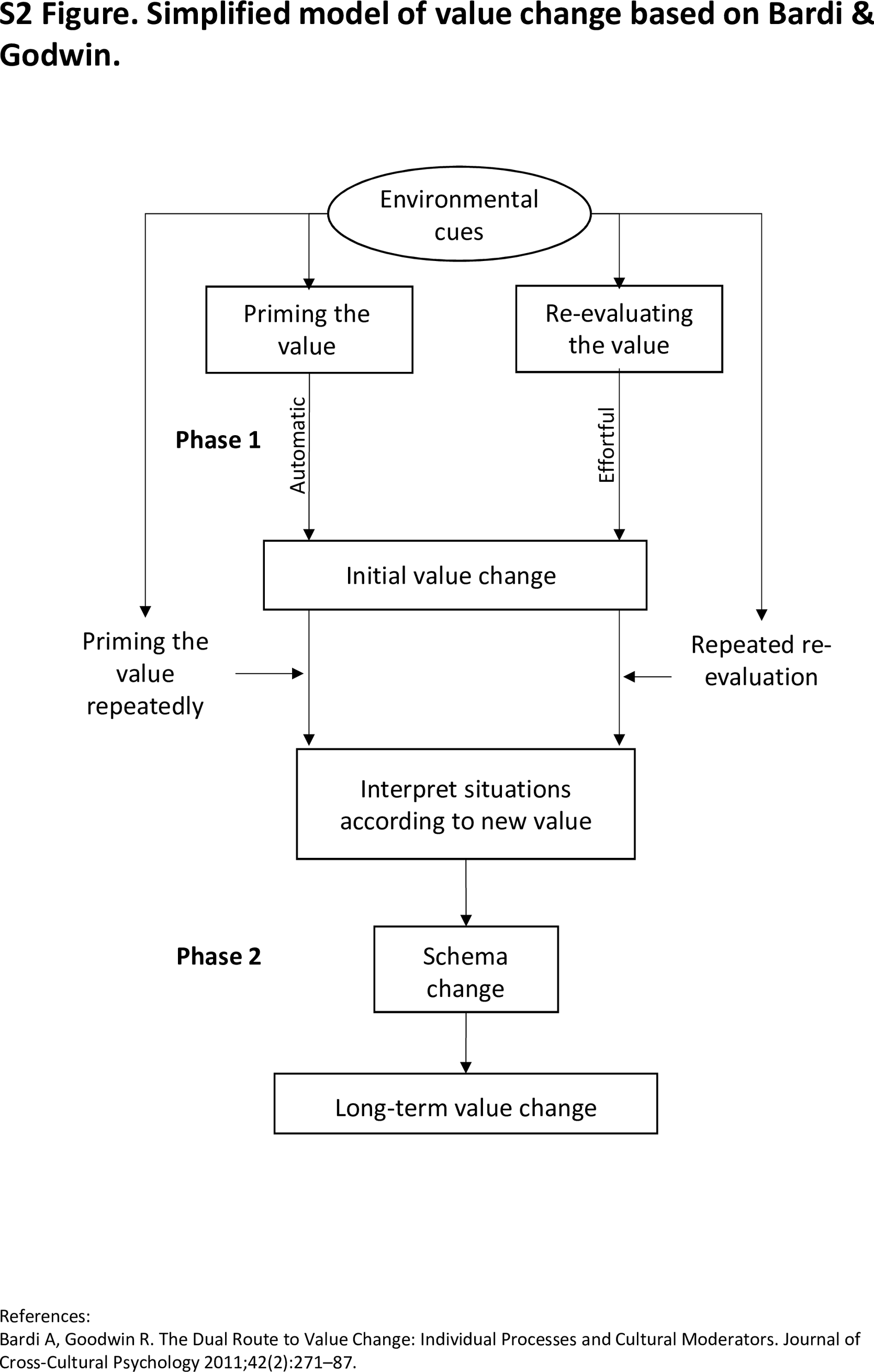

Supplement: S2 Fig — (TIF) [file pone.0297236.s002.tif]

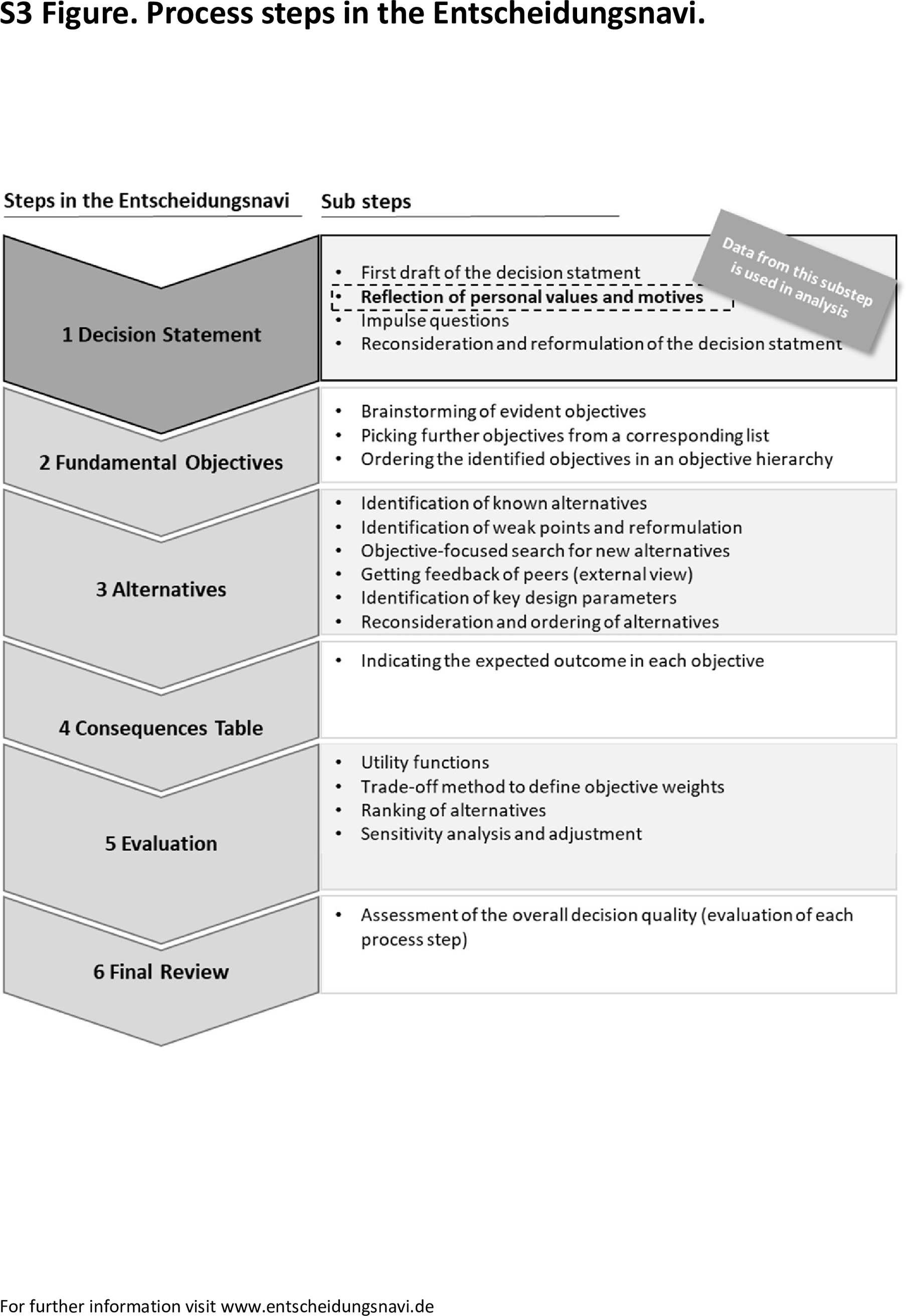

Supplement: S3 Fig — (TIF) [file pone.0297236.s003.tif]

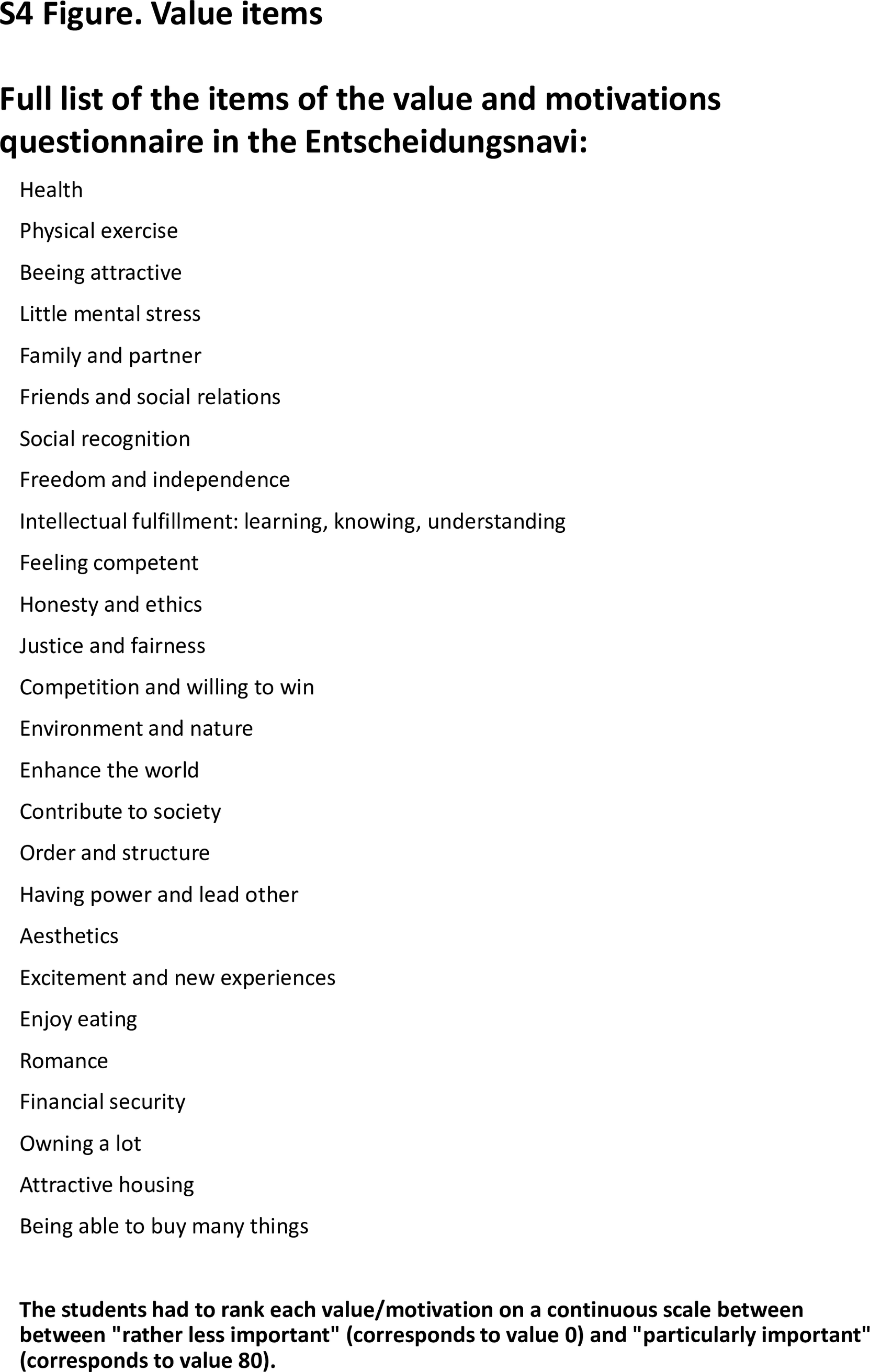

Supplement: S4 Fig — (TIF) [file pone.0297236.s004.tif]

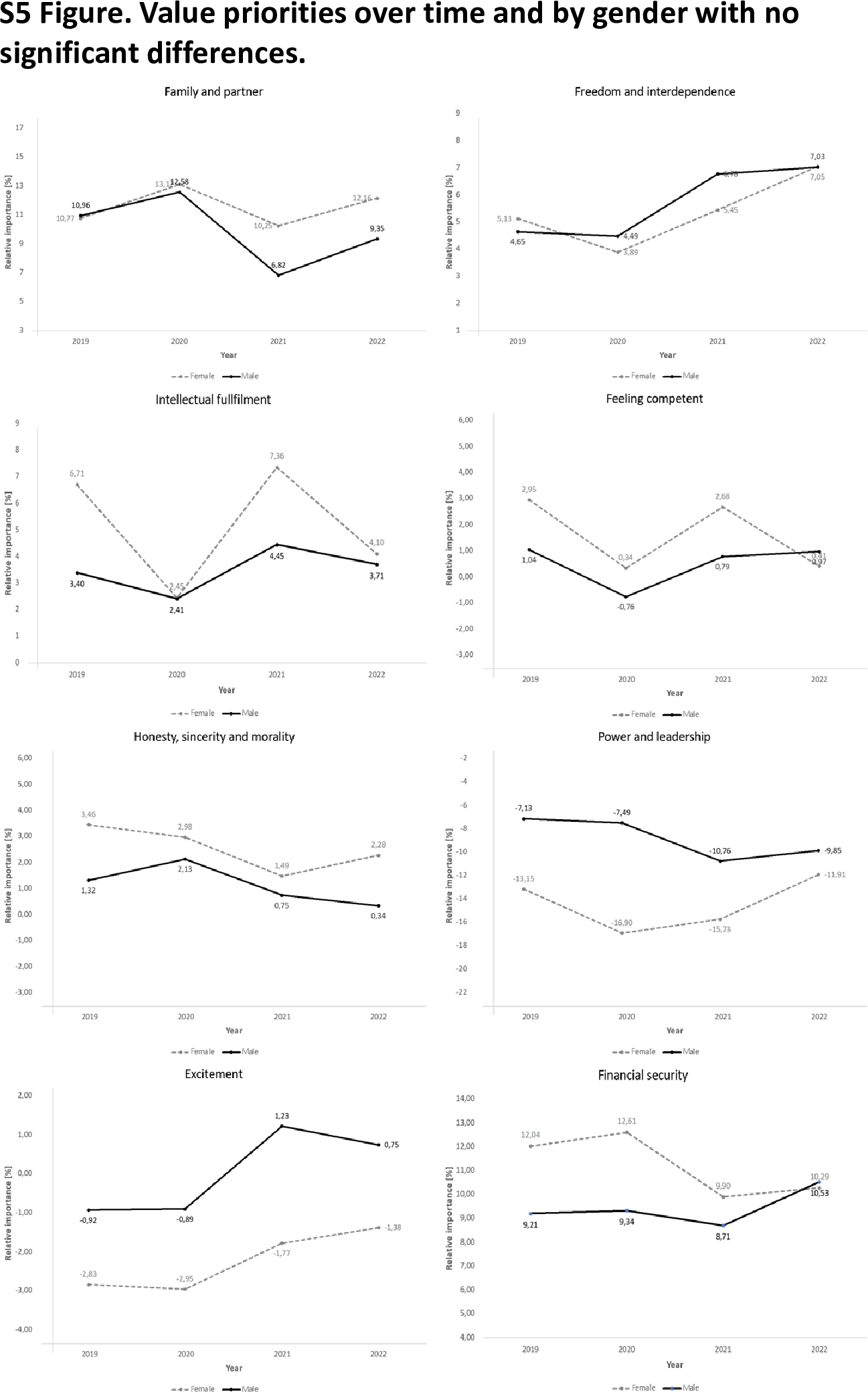

Supplement: S5 Fig — (TIF) [file pone.0297236.s005.tif]
